# Caspase-4 mediates cytoplasmic accumulation of TDP-43 in the primate brains

**DOI:** 10.1007/s00401-019-01979-0

**Published:** 2019-02-27

**Authors:** Peng Yin, Xiangyu Guo, Weili Yang, Sen Yan, Su Yang, Ting Zhao, Qiang Sun, Yunbo Liu, Shihua Li, Xiao-Jiang Li

**Affiliations:** 1 Ministry of Education CNS Regeneration Collaborative Joint Laboratory, Guangdong-Hongkong-Macau Institute of CNS Regeneration, Jinan University, Guangzhou, 510632 China; 2 Department of Human Genetics, Emory University School of Medicine, Atlanta, GA 30322 USA; 3 State Key Laboratory of Molecular Developmental Biology, Institute of Genetics and Developmental Biology, Chinese Academy of Sciences, Beijing, 100101 China; 4 Institute of Laboratory Animal Science, Chinese Academy of Medical Sciences and Peking Union Medical College, Beijing, 100021 China

**Keywords:** TDP-43, Caspase-4, Non-human primate, Aggregation, Neurodegeneration

## Abstract

The cytoplasmic accumulation of the nuclear TAR DNA-binding protein 43 (TDP-43) is a pathologic hallmark in amyotrophic lateral sclerosis, frontotemporal lobar degeneration, and other neurological disorders. However, most transgenic TDP-43 rodent models show predominant nuclear distribution of TDP-43 in the brain. By expressing mutant TDP-43 (M337V) in the brains of rhesus monkeys and mice, we verified that mutant TDP-43 is distributed in the cytoplasm of the monkey brain and that the majority of mutant TDP-43 remains in the nuclei of the mouse brain. The primate-specific caspase-4, but not mouse homologue caspase-11, could remove the NLS-containing N-terminal domain and generate fragmented TDP-43 that accumulates in the cytoplasm. Moreover, increased expression of caspase-4 in the monkey brain promotes the cytoplasmic accumulation of endogenous TDP-43, and suppressing caspase-4 reduces the cytoplasmic distribution of endogenous TDP-43 in cultured human neural cells. Our findings suggest that primate-specific caspase-4-mediated cleavage of TDP-43 accounts for its cytoplasmic mislocalization in the primate brains and may serve as a potential therapeutic target.

**Electronic supplementary material:**

The online version of this article (10.1007/s00401-019-01979-0) contains supplementary material, which is available to authorized users.

## Introduction

Amyotrophic lateral sclerosis (ALS) is a devastating neurodegenerative disease, characterized by muscle weakness due to the degeneration of large motor neurons. TAR DNA-binding protein of 43 kDa (TDP-43) accumulates and forms aggregates in the brains of patients with ALS and frontotemporal lobar degeneration (FTLD) [2, 7, 35]. Moreover, mutations in the TDP-43 gene are also associated with familial ALS and FTLD, indicating that mutant TDP-43 plays a causal role in neurodegeneration [1, 24, 45, 47]. TDP-43 is a nuclear protein of 414 amino acids consisting of two conserved RNA recognition motifs (RRM) and a C-terminal glycine-rich domain to associate with other heterogeneous ribonucleoprotein family members. It is involved in a variety of cellular functions including gene transcription, RNA processing, and protein homeostasis [12, 27, 39, 53]. Although TDP-43 carries the nuclear localization signals (NLS) in the N-terminal region and nuclear export signals (NES) in the middle region, it localizes in the cytoplasm to form aggregates in a variety of brain regions of patients with ALS, FTLD [5, 27, 35], and other pathological conditions [8, 9, 17, 23, 33, 48]. This cytoplasmic redistribution in human brains can lead to nuclear depletion of TDP-43 [2, 35], whereas cytoplasmic mislocalization of TDP-43 can cause a gain-of-toxicity [14, 19, 38]. Thus, the cytoplasmic mislocalization of TDP-43 plays an important role in the pathogenesis of a variety of neurodegenerative diseases.

The cytoplasmic distribution of mutant TDP-43 appears to be species dependent. This is because most transgenic mouse models of ALS show the predominant nuclear localization of transgenic TDP-43 [14, 19, 38, 43, 52, 59] and some mouse models only show the minimal level of cytoplasmic TDP-43 [34, 52, 55]. However, TDP-43 transgenic pig model displays the cytoplasmic distribution of TDP-43 [51]. This species-dependent phenomenon is not dependent on the level of TDP-43, as mice that overexpress transgenic TDP-43 or endogenously express mutant TDP-43 all show the predominant nuclear distribution of TDP-43 [19, 43, 46]. Since the cytoplasmic mislocalization of TDP-43 can lead to a gain-of-function in the cytoplasm and depletes its nuclear level to cause a loss-of-function in the nucleus, understanding how mutant TDP-43 accumulates in the cytoplasm is important for elucidating the pathogenesis of ALS, FTLD, and other neurological disorders. To this end, we compared the subcellular localization of mutant TDP-43 in the brains of mice and rhesus monkeys that were injected with the same viral vector-expressing mutant TDP-43. We found that the primate-specific caspase-4, but not mouse homologue caspase-11, is responsible for the generation of TDP-43 fragments that are able to accumulate in the cytoplasm. Furthermore, we found that endogenous TDP-43 in the monkey brain is also redistributed into the cytoplasm when caspase-4 is overexpressed and that suppressing caspase-4 can reduce the cytoplasmic distribution of endogenous TDP-43 in cultured human neural cells. Our findings suggest the caspase-4-mediated cytoplasmic accumulation of mutant TDP-43 is involved in ALS and other neurodegenerative diseases.

## Methods

### Plasmids, virus, antibodies, and reagents

The human mutant TDP-43(M337V) cDNA [51, 59] was subcloned into pAAV9-MCS or pGEX-4T1 vector (CellBiolabs) to generate AAV-mut-TDP-43(M337V) or GST-mut-TDP-43(M337V) vectors. The control AAV-GFP vector consisted of the same vector as for AAV-TDP-43 and contained the same ubiquitin promoter. Human caspase-4 or mouse caspase-11 cDNAs were generated using human or mouse brain tissues RNAs via RT-PCR, and cloned into pEGFP-C3, DesRed-C1, or AAV vector. AAV viruses (type-9) were packaged and amplified by the Viral Vector Core at Emory University. The titers of AAV vector genome were 1.4 × 10^13^ vg/ml for AAV-hTDP-43, 2.3 × 10^13^ vg/ml for AAV-GFP, and 1.8 × 10^13^ vg/ml for AAV-caspase-4. The following antibodies were used: mouse anti-human TDP-43 (Abnova, clone 2E2-D3) for the N-terminal TDP-43 (1-261 amino acids), rabbit anti-TDP-43 (Cell Signaling, G400) for a synthetic peptide corresponding to residues surrounding Gly400 of human TDP-43, rabbit anti-phospho-TDP-43 (Ser409/Ser410) (MABN14, Millipore), rabbit anti-LSD1 (2139S, Cell Signaling), rabbit anti-COX-IV (ab16056, Abcam), rabbit anti-PDI (2446S, Cell Signaling), rabbit anti-HSP90 (4874S, Cell Signaling), mouse anti-GFP (GTX628528, GeneTex), rabbit anti-NeuN (ABN78, Millipore), rabbit anti-GFAP (AB5804, Millipore), rabbit anti-caspase-4 (4450, Cell Signaling) and mouse anti-GST (Santa Cruz, A-6). All secondary antibodies were purchased from Jackson Immuno-Research Laboratories. Inhibitors of caspase family members included ZVAD-fmk (Selleckchem), DEVD-fmk (Tocris Bioscience), LEHD-fmk (Tocris Bioscience), and LEVD-fmk (BioVision).

### Ethics statement

Monkeys were housed in accordance with Chinese National standards, which are consistent with the standard set forth in the eighth edition of the NRC Guide for the Care and Use of Laboratory at the Institute of Laboratory Animal Science, Chinese Academy of Medical Sciences, Peking Union Medical College, which is fully accredited by the Association for Assessment and Accreditation of Laboratory Animal Care (AAALAC), International. The animal use and experiments followed the protocol that was approved by the Institutional Animal Care and Use Committee (IACUC) of the Institute of Laboratory Animal Science, Chinese Academy of Medical Sciences. This study was conducted in strict compliance with the “Guide for the Care and Use of Laboratory Animals of the Institute of Laboratory Animal Science (est. 2006)” and “The use of non-human primates in research of the Institute of Laboratory Animal Science (est. 2006)” to ensure the personnel safety and animal welfare.

All mice (C57BL/6) were bred and maintained in the animal facility at Emory University under specific pathogen-free conditions in accordance with institutional guidelines of the Animal Care and Use Committee at Emory University. The studies followed the protocol approved by the Animal Care and Use Committee at Emory University.

### Stereotaxic injection

Adult wildtype C57BL/6 mice at 8 months of age (*n* = 12 each group, six males and six females per group) were anesthetized by i.p. injection of 2.5% Avertin, and their heads were placed in a Kopf stereotaxic frame (Model 1900) equipped with a digital manipulator, a UMP3-1 Ultra pump, a 10 μl Hamilton microsyringe. A 33G needle was inserted through a 1 mm drill hole on the scalp. Injections occurred at the following stereotaxic coordinates: 3.1 mm posterior to bregma, 1.0 mm lateral to the midline, 4.7 mm ventral to the dura, with bregma set at zero. The microinjections were carried out at a rate of 0.2 μl/min. The microsyringe was left in place for an additional 10 min before and after each injection. 0.5 μl AAV-TDP43, AAV-caspase-4 or AAV-GFP virus was stereotaxically injected into the right substantia nigra of mice. Viral injection of monkey brains was performed using the facilities at Institute of Laboratory Animal Science, Chinese Academy of Medical Sciences, Peking Union Medical College.

For AAV viral injection into the monkey brain, we injected AAV-GFP or AAV-TDP-43 into the substantia nigra of male monkeys at the age of 8–12 years (*n* = 6 each group). Each monkey was anesthetized by intraperitoneal injection of 0.3–0.5 mg of atropine, followed by 10–12 mg of ketamine, and 15–20 mg of pelltobarbitalumnatricum per kg body weight. The monkeys were stabilized on a stereotaxic instrument (David Kopf). The precise position of substantia nigra for stereotaxic injection was located by MRI before injection. Ten μl viruses were injected at five different locations in the right substantia nigra (including three substantia nigra regions, pars compacta/SNpc, pars reticulate/SNpr, and pars lateralis/SNpl) in which the injection site was determined and the depth of needle insertion was calculated from the pre-operatively taken MRI. After the injection was given for 12 weeks, the monkeys with substantia nigra injections were subjected to behavioral analysis, and at the end of behavioral analysis, their brain tissues were isolated for immunohistochemical analysis. We also injected AAV-caspase-4 viruses (10 μl) into the prefrontal cortex of three monkeys. After 12 weeks, we isolated the injected brain tissues for studying the effect of caspase-4 on the cleavage of TDP-43.

### Mouse behavioral studies

All animal tests were performed in accordance with NIH guidelines for procedures and approved by the Institutional Animal Care and Use Committee of Emory University. Mouse behavior was assessed using a rotarod (Rotamex 4/8, Columbus Instruments International). Mice (C57BL/6) were trained for 10 min on three consecutive days with the rotarod speed at 5 rpm, and testing commenced after 3 days. The speed of the rod was set to 5 rpm and increased by 0.1 rpm/s. Each mouse went through three trials, and the average data of each group charted. The moribund mice were scored as “dead” and euthanized, and tissues were collected (*n* = 12 each group, six males and six females per group). The balance beam apparatus consists of 1 m beams with a flat surface of 12 mm or 6 mm width resting 50 cm above the table top on two poles. A black box at the end of the beam is the finish point. Nesting material from home cages in the black box serves to attract the mouse to the finish point. A lamp serves as an aversive stimulus, shining light above the start point. The time required for a mouse to cross to the center (80 cm) is measured by two motion detectors: one at 0 cm that starts a timer and one at 80 cm that stops the timer. The video camera records the performance.

### Monkey behavioral studies

Of six AAV-TDP-43- and six AAV-GFP-injected male monkeys at age of 8–12 years, four AAV-TDP-43- and two AAV-GFP-injected monkeys were selected, based on their similar body weights and ages, for behavioral analysis once each week for 12 weeks.

The movement capabilities of monkeys were tested as described previously [49]. Briefly, we carefully observed behavior of the monkeys during the day time and recorded video monitoring for 30 min per week. To evaluate weakness of the forelimb muscles on the AAV-injected monkeys, we performed the “open-field test” with continuous video recording. The “apple test” was performed and placed in line from back (monkey side) to front (observer side), to estimate the spontaneous action. In the “fence or grasping test”, we analyzed how frequently a monkey used his/her left or right hand to grasp the ceiling fence or test rod by video recording 30 min for each session each day. Four sessions were analyzed for obtaining the percentage of using the left or right hand. To evaluate weakness of the upper limb muscles, we measured grip strength using a spring hand dynamometer connecting with a small handle made specially for measuring monkey upper limb strength (AiDebaoHandPink, China). In this test, a monkey was allowed to grasp the handle with one upper limb (left or right), and its muscle strength was measured by the examiner pulling the spring hand dynamometer until the animal released the handle. The digital score of the force on the grip strength meter was recorded for statistical analysis.

### Human tissue acquisition

Human cortex tissues were obtained and archived via an institutional review board and Health Insurance Portability and Accountability Act compliant process at neuropathology/histochemistry core of Emory University. Autopsies occurred following death with a postmortem interval of 6–13 h. The cortex tissues were obtained from the postmortem brains of ALS patients (E04-56, E11-75, E09-35, E08-86 and E11-81) who died at 67–74 years of age and were confirmed to have TDP-43 aggregates via postmortem histologic analysis. Non-ALS control tissues were obtained from neurologically unaffected patients (E06-45, E06-114, E08-101, E08-137 and E10-142) who died at 53–92 years of age.

### Cell culture

Mouse neural crest-derived N2A cell line and human neuroblastoma SH-SY5Y cell line were purchased from ATCC and cultured in DMEM/F12 medium (containing 10% FBS, 100 U/ml penicillin, 100 μg/ml streptomycin, and 0.25 μg/ml amphotericin B). Medium was changed every 2 days. For culturing mouse primary neuronal cells, cortical neurons were isolated from the cortex of postnatal day 1 mice. Dissected tissue was treated with 0.0625 mg/ml trypsin and 0.0625 mg/ml DNasein 1 × HBSS buffer without calcium and magnesium for 10 min at 37 °C. Cells were washed once with the tissue culture medium, centrifuged at 1500 × *g* for 3 min, and then placed for initial growth in a 50% glial-conditioned medium (containing 0.25% glucose, 2 mM glutamate, 10% FCS, 500 nm insulin, 1 × vitamin mixture, and 1% antibiotic-antimycotic). The cells were cultured in neurobasal/B27 medium.

### TDP-43 expression studies

For analyzing TDP-43 expression and distribution in the monkey brain, we used three AAV-TDP-43 or AAV-GFP monkeys for Western blotting and another three AAV-TDP-43 or AAV-GFP monkeys for immunocytochemical studies. Animals were anesthetized and perfused with 10 ml 0.9% NaCl, and then with 20 ml of 4% paraformaldehyde in 0.1 M PBS through the left cardiac ventricle. Brains were removed and fixed overnight in the same solution and cryopreserved with 15% and 30% sucrose before sectioning into 10 µM sections with a cryostat (Leica CM1850) at − 20 °C. Sections from monkey or mouse brains or cultured cells were fixed in 4% paraformaldehyde in PBS for 10 min, permeabilized with 0.2% Triton X-100 in PBS for 30 min, blocked with 3% normal donkey serum in 3% BSA for 1 h, and incubated with primary antibodies in 3% BSA overnight at 4 °C. After several washes with PBS, the brain sections or fixed cells were incubated with secondary antibodies conjugated with either Alexa-488 or Alexa-594 (Invitrogen). 0.01 μg/ml DAPI was used to label the nuclei. Fluorescent images were taken with a Zeiss Axiovert 200 MOT microscope of the 40 ×/0.6 lens or 63 ×/0.75 lens, equipped with a digital camera (Hamamatsu, Orca-100) and Openlab software (Improvision). The immunostaining analysis of TDP-43 subcellular distribution in the injected monkey or mouse brains was performed completely blinded on standardized 40 mm sections. The monkey brain sections were prepared using a brain slicer including the injected regions (3 substantia nigra: pars compacta/SNpc, pars reticulate/SNpr and pars lateralis/SNpl). Each brain region was used to take at least six images (40 × magnification) that can clearly reveal the subcellular distribution of TDP-43. For the quantitative analysis of differential subcellular location of TDP-43 in the monkey and mouse brain, the numbers of cells showing the nuclear or cytoplasmic TDP-43 per image were presented as the mean ± SEM, and the quantitative data were obtained from three monkeys or six mice per group. Densitometry analyses of fluorescent intensities of aggregates were quantified by ImageJ software (W. Rasband, National Institutes of health, USA).

### Subcellular fractionations of brain tissues

Monkey or mouse brain tissues were homogenized for 25 strokes with a dounce homogenizer ice-cold buffer (0.32 M sucrose, 15 mM Tris–HCl, 60 mM KCl, 15 mM NaCl, 5 mM EDTA, 1 mM EGTA, 0.02% NaN_3_, 2 mM ATP, pH 8.0) containing protease inhibitor (Roche) and 100 μM PMSF. Ten percent lysates were stored as the total lysate sample. Nuclei and cellular debris were pelleted (P1) at 800 × *g* for 5 min. The supernatant (S1) was transferred to a new tube and centrifuged at 20,000 × *g* for 30 min at 4 °C to obtain the mitochondria-enriched pellet (P2). The supernatant (S2) was then used for the soluble cytoplasmic fraction. The S2 was centrifuged at 100,000 × *g* for 30 min at 4 °C to obtain the endoplasmic reticulum-enriched pellet (P3). Crude nuclear pellets were washed four times with ice-cold homogenization buffer to remove cytoplasmic contaminants. For nuclear purification, the pellets were re-suspended in 374 μl of buffer [15 mM HEPES, 1.5 mM MgCl_2_, 0.2 mM EDTA, 0.5 mM DTT, 26% glycerol (v/v), pH 7.9] with 26 μl of 4.6 M NaCl to generate the final concentration at 300 mM NaCl, homogenized with 20 full strokes in Teflon homogenizer on ice, and sonicated for 10 s. The homogenized samples were kept on ice for 20 min and then centrifuged at 24,000 × *g* for 20 min at 4 °C.

### Caspase-4 activity assay

All the tissue samples were adjusted to 0.5 mg/ml total protein by dilution with homogenization buffer for triplicate caspase assays. Caspase-4 activity was determined using the specific Ac-YVAD-AFC substrate (10 μg/ml; BioVision). Equal amounts (10 μg) of the extracts were incubated with corresponding substrates in 100 μl caspase-4 activity assay buffer (0.05 M Tris–HCl, 0.5 mM EDTA, 1 mM ATP, 1 mM DTT, pH8.0) for 30–60 min at 37 °C. Cold water (0.8 ml) was used to stop the reactions, and the reaction mixtures were iced for at least 10 min. The free AFC fluorescence was quantified using the CytoFluor multi-well plate reader (FLUOstar; BMG LABTECH) with excitation and emission wavelengths at 400 nm and 500 nm, respectively. All readings were standardized using the fluorescence intensity of an equal volume of free 7-amino-4-trifluoromethylcoumarin (AFC) solution (40 mM), normalized by the protein concentrations and expressed as nmol/min/mg protein.

### In vitro caspase assay

Purified GST-mut-TDP-43 (M337V) in Sepharose beads were diluted in cold assay buffer (25 mM Tris–HCl, 10 mM MgCl_2_, 100 µg/ml purified rabbit creatine kinase, 50 mM phosphocreatine, 1 mM ATP, pH 7.6). Monkey or mouse tissues form the brain striatum and cortex were homogenized at 1 g/ml in cold assay buffer using 20 strokes of a glass dounce hand homogenizer and were centrifuged at 500 × *g* for 5 min at 4 °C to pellet unbroken tissues and membranes. The supernatant was collected and stored on ice, while protein concentrations were determined using a BCA Protein Assay Kit (Thermo Scientific). The lysates (200 μl) at 0.5 mg protein/ml were incubated with GST-TDP-43 beads (200 μl) at 37 °C with 300 rpm shaking for 24 h. The beads were centrifuged and combined with the protein loading dye (0.2% SDS) for SDS-PAGE and Western blotting analysis to detect expression of the cleaved GST-TDP-43 using an anti-GST antibody. An anti-C-terminal TDP-43 antibody was used to detect the presence of C-terminal TDP-43 in the supernatant. Different caspase inhibitors at the concentrations (50–100 μM) were included in the lysates to inhibit caspase activity during the incubation process with GST-TDP-43 beads.

### Transfection of cultured cells

Cultured cells were transfected with plasmid DNAs using RNAi Max transfection reagent (Invitrogen) according to the manufacturer’s protocol. At 48 h following transfection, cells were harvested for immunofluorescent staining and Western blotting. For caspase-4 knockdown experiment, the cultured human neural SH-SY5Y cells were transiently transfected with caspase-4 siRNA (Gene Pham Co. sequence GUGUAGAUGUAGAAGAGAAtt or AAGUGGCCUCUUCACAGUCAUtt) or control siRNA (scrambled sequence) using RNAi Max transfection reagent (Invitrogen) according to the manufacturer’s protocol. At 48 h following transfection, cells were harvested for immunofluorescent staining and Western blotting.

### Cell viability assay

The cell viability assay was done using Cell Counting Kit-8 (CCK-8) (Dojindo, Japan), which determines the number of viable cells in proliferation and cytotoxicity. It utilizes tetrazolium-8-[2-(2-methoxy-4-nitrophenyl)-3-(4-nitrophenyl)-5-(2,4-disulfophenyl)-2H-tetrazolium] monosodium salt that produces a water-soluble formazan dye in living cells. The amount of this dye is directly proportional to the number of living cells, offering a sensitive assay to detect viability of many cell lines including SH-SY5Y cells [10, 56]. Briefly, SH-SY5Y cells were transfected with siRNA for 48 h and then treated with tunicamycin (1 μg/ml) for 12 h. An equal number of 100 µl SH-SY5Y cell suspension (1 × 10^4^ cells/100 µl/well) was dispensed in a 96-well plate. Each well of the plate was added with 10 µl CCK-8 solution, and incubated for 2 h at 37 °C. The absorbance values at the wavelength of 450 nm were spectrophotometrically measured using an CytoFluor multi-well plate reader (FLUOstar; BMG LABTECH). Each sample was tested in three replicates. SH-SY5Y cells without transfection and tunicamycin treatment were used as negative and blank controls. The values (mean ± SEM) of treated SH-SY5Y cells were calculated as  % of control cells.

### Statistical analysis

Statistical significance was assessed using the two-tailed Student’s *t* test for comparing two groups. When analyzing multiple groups, we used one-way ANOVA to determine statistical significance. For mice or monkey that was repeatedly subjected to behavioral tests, we analyzed the data using two-way ANOVA. Data are mean ± SEM. Calculations were performed with GraphPad Prism software.

## Results

### Expression of mutant TDP-43 and the associated phenotypes in the brains of mice and monkeys

**Fig. 1:**
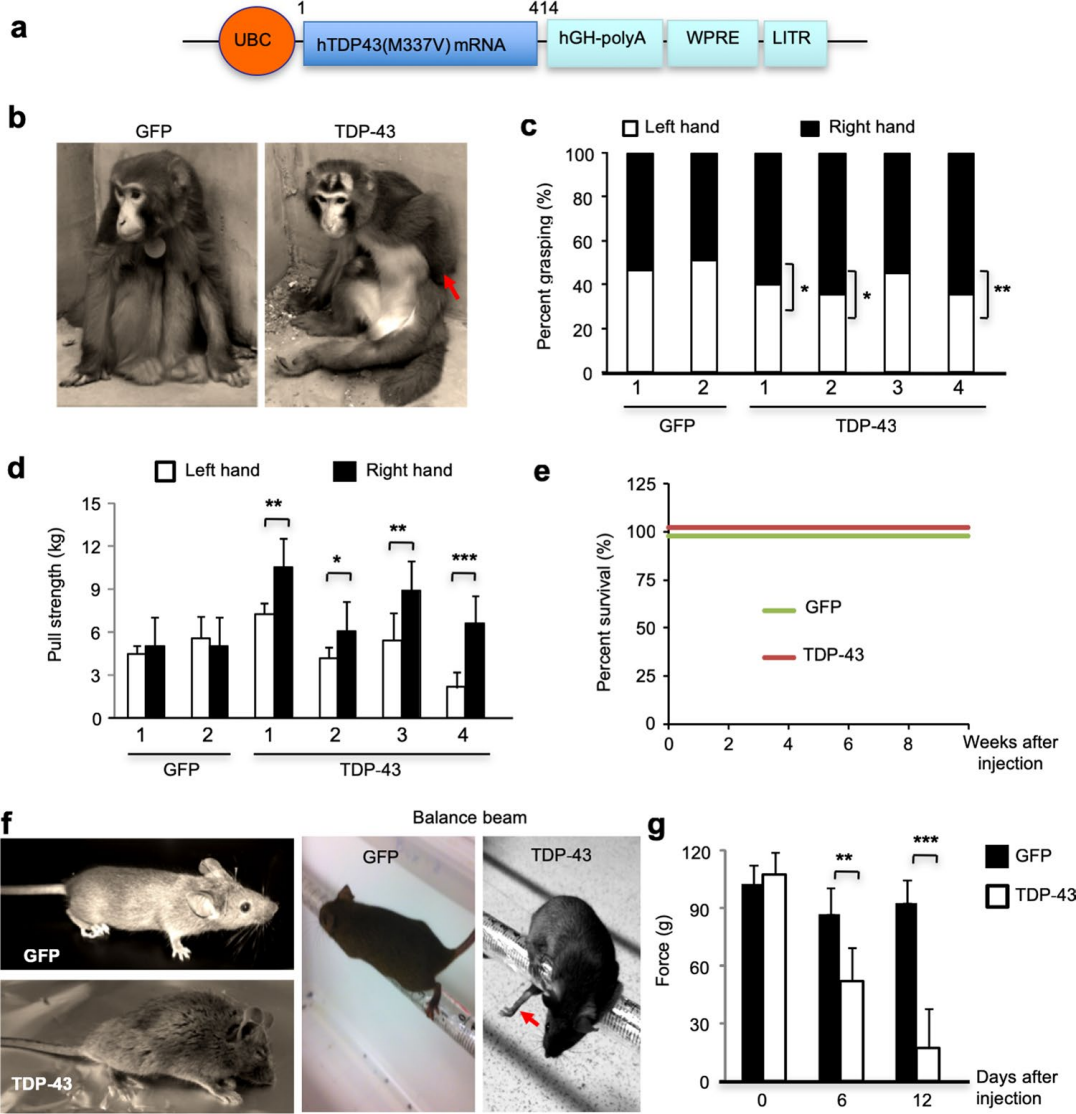
Phenotypes of AAV-TDP-43-injected monkeys and mice. **a** Adeno-associated virus vector-expressing mutant TDP-43(M337V) under the control of the human ubiquitin promoter. **b** Photographs of representative monkeys that were injected with AAV-GFP (control) or AAV-TDP-43 in the right side of the substantia nigra. The AAV-TDP-43-injected monkey showed the paralyzed left upper limb (arrow), whose movement is controlled by the contralateral right side of the substantia nigra. **c** AAV-TDP-43-injected monkeys showing decreased grasping by the left upper limb. Four AAV-TDP-43-injected and two AAV-GFP-injected monkeys were video recorded for the hand (left and right upper limbs) grasping of the ceiling fence and a test rod. Each monkey was video recorded for 30 min each time. The numbers of grasping from six recording times were used for statistical analysis (*n* = 6 times on the same examining day, mean ± SEM, *P < 0.05, **P < 0.01). **d** AAV-TDP-43-injected monkey showing decreased grip strength of the left upper limb. Monkeys in (**c**) were measured for the grip strength of their left and right hands by pulling a spring hand dynamometer. Each monkey was tested six times on the same examining day, and the digital score of the force (kg) on the grip strength meter was recorded for analysis (*n* = 6 times, mean ± SEM, *P < 0.05, **P < 0.01, ***P < 0.001). **e** Survival plot showing that expressing TDP-43 in the substantia nigra did not affect the monkey survival rate. The monkeys at 9–15 years of age were injected with either AAV-GFP or AAV-TDP-43 (*n* = 6 monkeys in each group). **f** Photographs of a representative mouse expressing AAV-TDP-43 (10^8^ vg) in the right side of substantia nigra and showing the hunchback phenotype and defective balance beam movement (arrow). An AAV-GFP (10^8^ vg)-injected mouse served as a control. **g** Force tests showing that injection of AAV-TDP-43 in the substantia nigra of mice caused motor function impairment, which was revealed by hind limb grip strength assay. AAV-GFP injection served as a control. Mice were 8–12 months old and injected with AAV-TDP-43 or AAV-GFP (*n* = 12 mice, mean ± SEM, in each group, **P < 0.01, ***P < 0.001)

Our previous studies demonstrated that stereotaxic injection of mutant TDP-43 in the striatum of mice led to abundant nuclear TDP-43 in the injected brain [59]. Using an adenoviral-associated vector (AAV) that expresses human TDP-43 (M337V) under the control of the ubiquitin promoter (Fig. 1a) and the same stereotaxic approaches in our previous study [60], we injected AAV-TDP-43 into the right substantia nigra of six male rhesus monkeys (8–12 years) and twelve mice (10–14 months). In our experiments, we used the substantia nigra in the monkey because this small brain region is suitable for viral injection and controls the movements of the limbs and elbows [31, 54], allowing us to examine the subcellular distribution of TDP-43 as well as the phenotypes associated with TDP-43 toxicity. Upper limb and elbow movements were measured to determine the effects in the animals. The controls included the injection of the same AAV vector-expressing GFP (AAV-GFP).

Three months after the injection, the AAV-GFP monkeys did not show any motor symptoms, but all the TDP-43-injected monkeys developed the obvious weakness of the left upper limb, whose movement is controlled by the contralateral right substantia nigra that was injected with AAV-TDP-43 (Fig. 1b). These upper limb weaknesses appeared 2–4 weeks post injection, increased in severity, and stabilized after 3–4 months. Daily video showed that TDP-43-injected monkeys displayed significantly infrequent use of their left upper limbs as compared with the GFP-injected control monkeys when walking and grasping food, the ceiling fence, or a rod (Suppl. Figure 1; Suppl. Video.1). Because these monkeys could not raise left upper limbs and straighten left elbow joints, they had difficulties reaching and grasping the celling fence or a test rod (Fig. 1c). Some monkeys had paralyzed upper limbs, resulting in walking difficulties. By measuring the grip strength using a spring hand dynamometer, we found that TDP-43-injected monkeys had a significant decrease in the left upper limb muscle strength compared with the right upper limb (Fig. 1d). The severe limb movement impairment could be due to the acute and broad toxicity of overexpressed TDP-43 in the injected substantia nigra. However, no early death occurred in all monkeys injected with AAV-TDP-43 (Fig. 1e). Similarly, overexpressing TDP-43 (M337V) in the right substantia nigra in mice also led to markedly reduced grip strength of the left limb (Fig. 1f, g) and poor rotarod and balance beam performance within 2 weeks after viral injection (Suppl. Figure 2).

### Differential subcellular accumulation of mutant TDP-43 in the brains of monkeys and mice

**Fig. 2:**
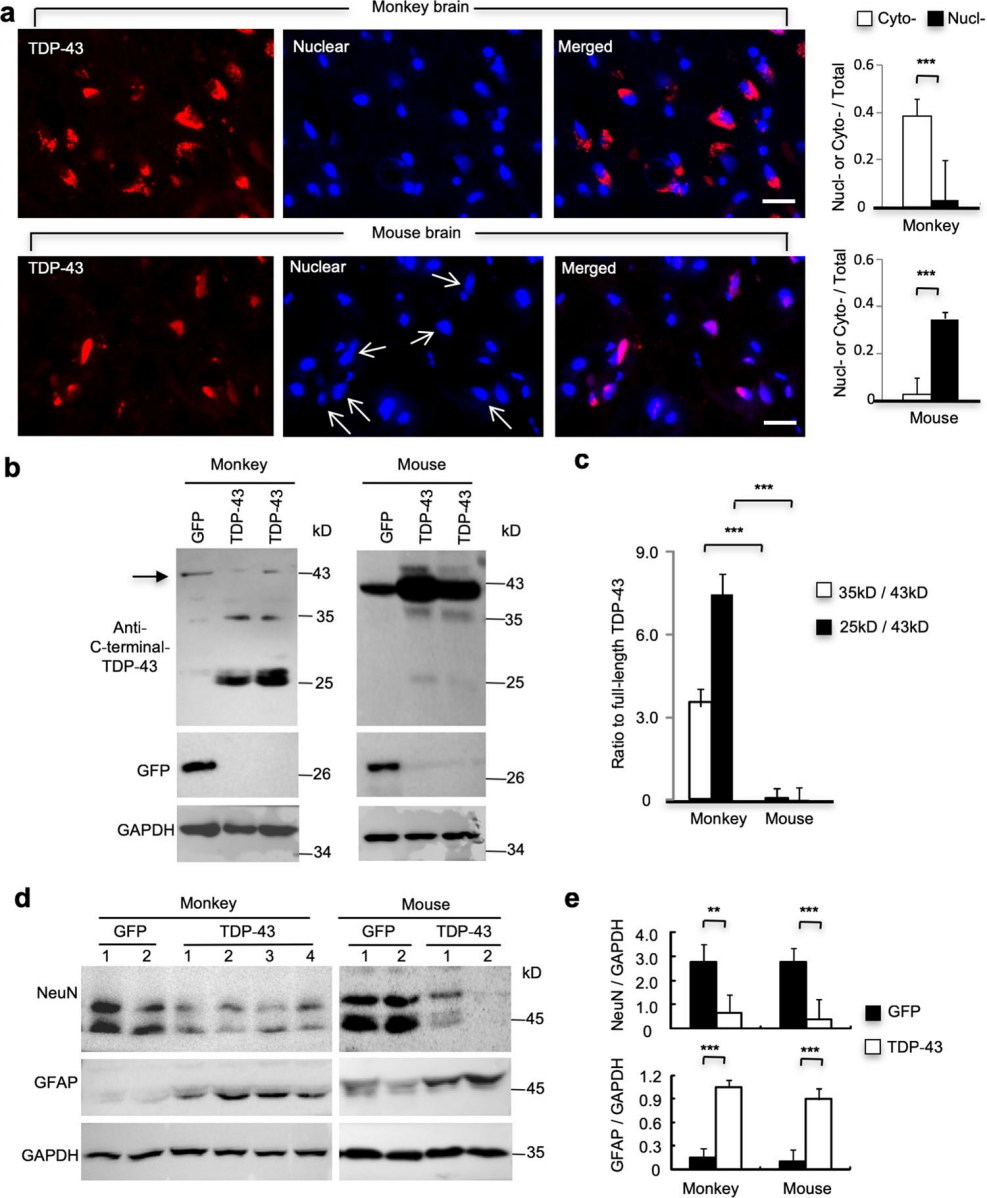
Differential subcellular location and degradation of mutant TDP-43 (M337V) in the monkey and mouse brains. **a** TDP-43 immunostaining of the monkey substantia nigra showing the cytoplasmic localization of mutant TDP-43 (red, upper panel) after AAV-TDP-43 injection. The nuclei of neurons were labeled by DAPI (blue). In the mouse brain (lower panel), mutant TDP-43 was predominantly localized in the nuclei (arrows) of the injected substantia nigra. The relative numbers of cells containing nuclear (Nucl) or cytoplasmic (Cyto) TDP-43 over total DAPI-staining cells are shown in the right panel (mean ± SEM, ***P < 0.001). Scale bar 20 μm. **b** Western blotting analysis of the brain substantia nigra tissues of AAV-TDP-43-injected monkeys and mice. Probing with an antibody to C-terminal-TDP-43 revealed that C-terminal TDP-43 fragments (35- and 25-kD) were abundant in the monkey tissues but were weakly seen in the injected mouse brain. **c** Ratios of truncated TDP-43 (35- and 25-kD) to full-length TDP-43 detected by Western blotting in (**b**). The ratios were obtained from three independent Western blotting experiments (***P < 0.001). **d** Western blotting analysis of the expression of neuronal (NeuN) and astrocytic (GFAP) proteins in the AAV-GFP- or AAV-TDP-43-injected substantia nigra of monkeys and mice. Western blots were probed with antibodies to NeuN, GFAP, and GAPDH. **e** Ratios of NeuN/GAPDH and GFAP/GAPDH on the Western blotting in (**d**). The data were obtained from three independent Western blotting analysis of two AAV-GFP- and two AAV-TDP-43-injected mouse substantia nigra tissues; or two AAV-GFP- and four AAV-TDP-43-injected monkey substantia nigra tissues (**P < 0.01; ***P < 0.001)

The neurological phenotypes of the TDP-43-injected mice and monkeys confirmed the neurotoxicity of overexpressed mutant TDP-43 and also allowed us to compare TDP-43’s expression and localization in these two different species. The brains of mice and monkeys were dissected after euthanization and prepared for Western blotting and immunocytochemistry. We used an antibody against a C-terminal peptide corresponding to residues surrounding Gly400 of human TDP-43 to detect the overexpressed exogenous TDP-43. Immunocytochemistry revealed that injection of the control AAV-GFP yielded diffuse green fluorescence throughout the entire cell (Suppl. Figure 3). However, the injected mutant TDP-43 was diffusely distributed in the cytoplasm or adjacent to the nuclei in the monkey brain (Fig. 2a). In contrast, the injected mouse brain showed the predominant nuclear distribution of mutant TDP-43 (Fig. 2a), similar to the abundant nuclear localization of transgenic TDP-43 found in different mouse models [14, 19, 43, 52, 59]. Quantification of the percentage of cells showing cytoplasmic or nuclear accumulation of transgenic TDP-43 in the injected brain region also verified the remarkable differences in the subcellular distribution of mutant TDP-43 in the mouse and monkey brains (Fig. 2a).

### Differential cleavage of TDP-43 in the monkey and mouse brains

The above findings have shown that different subcellular localization of TDP-43 is species-dependent even when mutant TDP-43 is overexpressed. To investigate whether there is any difference in TDP-43 cleavage, we performed Western blotting using anti-C-terminal TDP-43 antibody. Interestingly, truncated TDP-43 fragments (35- and 25-kD) were abundant in the monkey brain, whereas full-length TDP-43 is expressed at a comparable level as endogenous TDP-43 in the AAV-GFP-injected monkey. However, in the mouse brain, full-length TDP-43 was much more abundant than C-terminal truncated TDP-43, which is consistent with the previous findings from transgenic TDP-43 mice [34, 52, 55]. The low level of truncated TDP-43 in the mouse brain is remarkably different from the abundant level of truncated TDP-43 in the monkey brain (Fig. 2b). The quantification of the relative level of intact and truncated TDP-43 on Western blotting also verified significant differences in TDP-43 cleavage in monkey and mouse brains (Fig. 2c). Western blotting revealed a significant reduction of the neuronal marker NeuN and an increase in GFAP staining, which reflects neuronal toxicity and reactive astrocytes, in both mouse and monkey brains (Fig. 2d, e). Double immunostaining verified the reduced number of NeuN-positive cells in AAV-TDP-43 injected monkey brain region (Suppl. Figure 4), which was similar to what we found in the AAV-TDP-43 injected mouse brain [59]. These alternations indicate neurodegeneration in the injected brain regions, and are consistent with the phenotypes of the injected monkeys and mice. Using an antibody to N-terminal TDP-43 (1-261 amino acids) or phosphorylated S409/S410 TDP-43, we also found that TDP-43 is readily cleaved in the monkey brain, but not in the mouse brain (Suppl. Figure 5). These findings suggest that monkey brain tissues can selectively cleave TDP-43 to generate C-terminal TDP-43 fragments, which may accumulate in the cytoplasm.

**Fig. 3:**
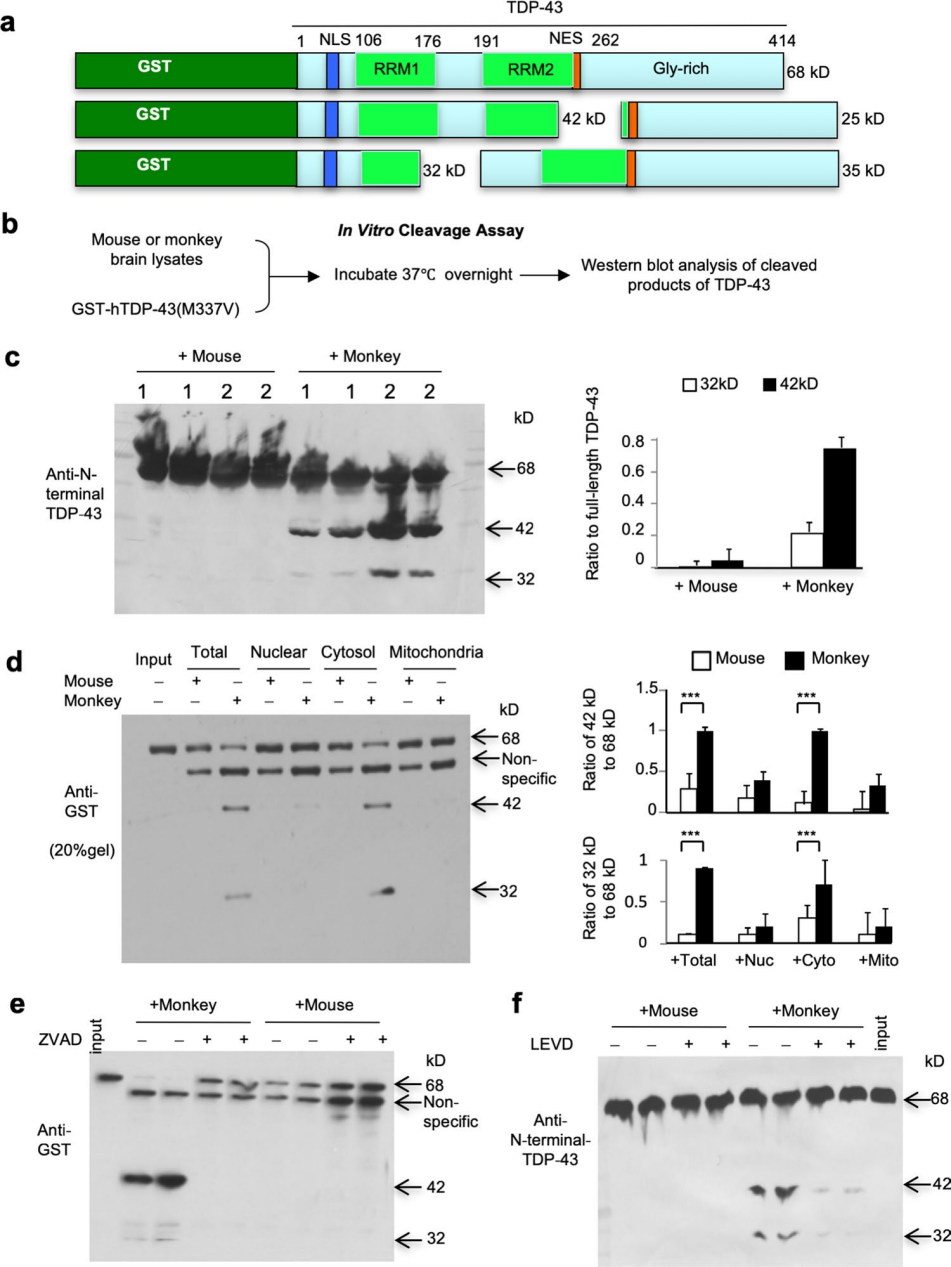
Caspase-4 selectively cleaves TDP-43 in vitro. **a** The plasmid DNA structures for expressing GST-TDP-43 fusion protein. The cleavage of TDP-43 (35- and 25-kD) seen in the monkey brain can generate truncated GST-TDP-43 fusion proteins at 42- and 32-kD, respectively. The intact GST-TDP-43 is about 68-kD in size. RRM: RNA recognition motifs. **b** In vitro caspase assay of TDP-43. GST fusion proteins containing mutant TDP-43(M337V) in Sepharose beads were incubated overnight with the same quantity of mouse or monkey brain lysates. The beads were then centrifuged and analyzed by Western blotting with anti-GST. **c** Western blotting analysis of GST-TDP-43 that was incubated with brain homogenate lysates from monkeys or mice. The beads were centrifuged and analyzed with anti-GST. Expression levels of these fragments were minimal when incubated with mouse brain lysates. The ratios of fragmented TDP-43 to the intact full-length GST-TDP-43 (68-kD) were obtained from three independent experiments and shown in the right panel. **d** Western blotting analysis of GST-TDP-43(M337V) cleavage after incubation with different subcellular fractions (total homogenate, nuclear, cytosol, and mitochondria) of the mouse or monkey brains. The cytosolic fraction generated more fragmented TDP-43 (42-kD and 32-kD) than other fractions. Ratios of the 42-kD or 32-kD band to the intact GST-TDP-43 (68-kD) are presented in the right panel. The data are mean ± SEM (*n* = 3, ***P < 0.001). **e, f** Western blotting analysis of GST-mutant-TDP-43(M337V) cleavage showing that incubation of the pan-caspase inhibitor ZVAD-fmk (**e**) or the caspase-4 inhibitor LEVD-fmk (**f**) was able to block TDP-43 cleavage by the monkey brain lysates. The input lane is aliquot GST-TDP-43 (68-kD) without incubation with monkey or mice tissue lysates

The above striking difference led us to examine the differential processing of TDP-43 in the monkey and mouse brains. TDP-43 consists of 414 amino acids with NLS in the N-terminal region and NES in the region close to the C-terminus (Fig. 3a, Suppl. Figure 6). We generated PRK vectors to express N-terminal fragments (1–173), truncated (77–414), C-terminal (174–414), or full-length TDP-43 under the CMV promoter (Suppl. Figure 6). The expression of these TDP-43 proteins in transfected N2A cells was detected using Western blotting and immunostaining with antibodies that recognized C-terminal or N-terminal TDP-43. Both truncated (77–414) and C-terminal (174–414) fragments of TDP-43 were distributed in the cytoplasmic region. In contrast, N-terminal (1-173) TDP-43 with the NLS signal sequences is localized in the nucleus (Suppl. Figure 7). These results suggest that the specific processing of TDP-43 in the monkey brains may generate TDP-43 fragments that lack N-terminal NLS and are able to accumulate in the cytoplasm.

To test the above idea, we generated recombinant GST fusion proteins containing mutant TDP-43 (M337V) that was produced in bacteria and then purified (Fig. 3a). Purified GST-TDP-43 proteins in glutathione-affinity beads were incubated overnight with the monkey or mouse brain lysates at 37 °C for in vitro cleavage. Because of the limited substantia nigra tissues, we used the striatum and cortex tissue lysates from monkeys and mice in this in vitro cleavage assay. The beads were precipitated and immuno-blotted by anti-GST to detect cleaved GST-TDP-43 products (Fig. 3b). This in vitro analysis revealed that monkey brain tissues could cleave full-length TDP-43 to generate fragments (32- and 42-kD) that were detected by anti-GST and anti-N-terminal TDP-43 (Fig. 3c). Generation of these GST fusion protein fragments corresponds to the production of cleaved C-terminal TDP-43 products (25- and 35-kD) (Fig. 3a), which are present in the monkey brain (Fig. 2b, c) and in the in vitro cleavage assay (Suppl. Figure 8). Using different subcellular fractions from monkey and mouse brain tissues to incubate with full-length GST-TDP-43 (Suppl. Figure 9), we found that the cytosolic fraction had the greatest activity to generate fragmented TDP-43 than the nuclear and mitochondrial fractions (Fig. 3d), suggesting that TDP-43 is cleaved by cytosolic molecules in the monkey brains. We then applied MG132 and BFA to inhibit the ubiquitin–proteasome system and autophagy, respectively, and found that these inhibitors did not abolish the generation of TDP-43 fragments but could increase the levels of fragmented TDP-43 produced by monkey brain lysates (Suppl. Figure 10a). These findings suggest that a specific cytosolic enzyme rather than the UPS and autophagy in the monkey brain may generate TDP-43 fragments, which are then degraded by the UPS or autophagy.

### Caspase-4 is important for generating TDP-43 fragments

In vitro experiments revealed that multiple caspases (caspases-3, -4, -7 and -9) cleave TDP-43 in cultured cells [6, 11, 26, 29, 42]. However, these in vitro experiments introduced caspases by transfection into cultured cells and did not tell us whether some of these caspases can endogenously determine the specific cleavage of TDP-43 in the primate brains. To investigate whether caspases generate the specific cleavage products of TDP-43 in the monkey brain, we first used the pan-caspase inhibitor, ZVAD-fmk to inhibit all the caspase family members and found that this inhibitor completely blocked the generation of fragmented TDP-43 by the monkey brain lysates (Fig. 3e). Since the in vitro study demonstrated that TDP-43 was initially cleaved after Asp174 by caspase-4 [29], we added the caspase-4-specific inhibitor LEVD-fmk and found that it could effectively block the cleavage of TDP-43 and prevent the generation of 42- and 32-kD fragments (Fig. 3f). However, neither of DEVD-fmk inhibitors of caspase-3 and -7, nor LEHD-fmk inhibitor of caspase-9 could prevent the initial cleavage of TDP-43 (Suppl. Figure 10b, c).

**Fig. 4:**
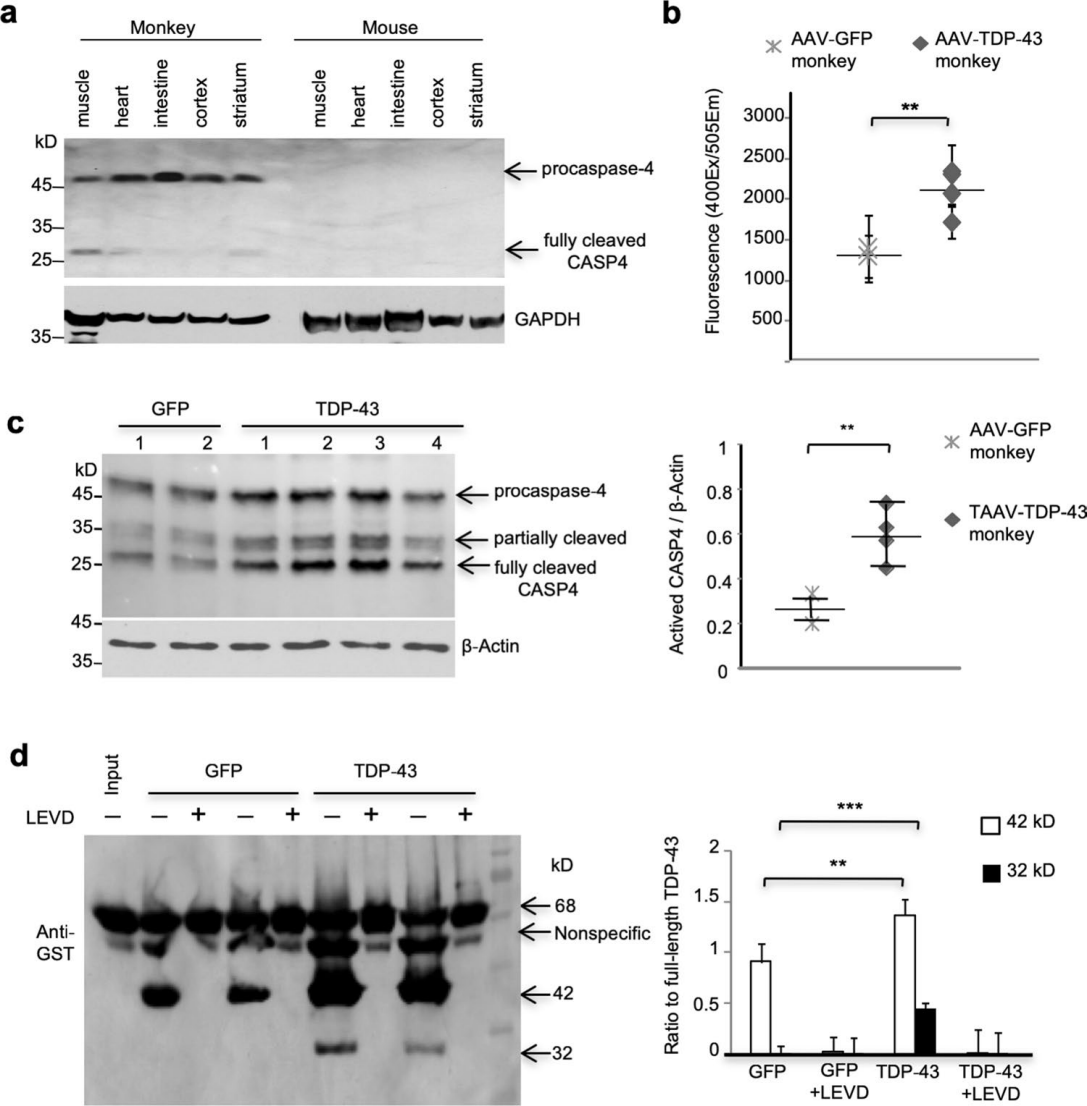
Caspase-4 is specifically expressed in the monkey brain and its activity can be up-regulated by mutant TDP-43. **a** Western blotting analysis of caspase-4 (CASP4) expression in the brain and peripheral tissues of wildtype monkeys and mice. **b** Caspase-4 (CASP4) activity in AAV-GFP- or AAV-TDP-43(M337V)-injected monkey brain was assayed using a fluorescence method with a specific caspase-4 substrate Ac-YVAD-AFC. The data are mean ± SEM (*n* = 3 AAV-GFP monkeys, and *n* = 3 AAV-TDP-43 monkeys, **P < 0.01). **c** Western blotting analysis of caspase-4 expression in AAV-GFP- or AAV-TDP-43(M337V)-injected monkey brain. The activated form of caspase-4 (arrow) is more abundant in AAV-TDP-43-injected brain region than AAV-GFP-injected monkey brain. Quantitative data (ratio of activated caspase-4 to β-Actin) are shown in the right panel from three independent experiments (**P < 0.01). **d** Western blotting analysis of GST-TDP-43(M337V) incubated with lysates from AAV-GFP- or AAV-TDP-43-injected monkey brains. More cleaved GST-TDP-43 fragments were found in the AAV-TDP-43-injected brain lysates, and the caspase-4 inhibitor LEVD-fmk could completely block the cleavage. Quantitative data of the ratios of cleaved TDP-43 fragments to the intact form (68 kD) are shown in the right panel from three independent experiments (**P < 0.01, ***P < 0.001)

Caspase-4 is an enzyme that cleaves proteins proteolytically at an aspartic acid residue and contributes to inflammatory activation in mono-myelocytic cells [4, 44, 50]. Interestingly, by analyzing amino acid sequences of human caspase-4 and its homologues in monkey and mouse, we found that caspase-4 is only present in primates and its rodent homologue is caspase-11. Caspase-4 in humans and monkeys share up to 95% identify in amino acid sequences, but human caspase-4 shares only 50% identify with mouse caspase-11 (Suppl. Figure 11). Immuno-blotting with the antibody to human caspase-4 showed that caspase-4 is widely present in different brain regions and peripheral tissues in monkeys, while the anti-caspase-4 failed to detect any band in the mouse tissues (Fig. 4a).

Caspase-4 is bound to the endoplasmic reticulum (ER) and can be activated under ER stress [18]. Previous reports note that overexpression of TDP-43 or other misfolded proteins induces ER stress [32, 36, 37]. A fluorescence assay using caspase-4 substrate Ac-YVAD-AFC indeed revealed elevated caspase-4 activity in TDP-43-injected monkey brain as compared with GFP-injected monkey (Fig. 4b). The cleaved and activated caspase-4 (between 25- and 45-kD) were also up-regulated in the TDP-43-injected monkey substantia nigra as compared with the control GFP-injected monkey (Fig. 4c). This finding explains the data in Fig. 2B that shows more abundant cleaved TDP-43 fragments than full-length mutant TDP-43 in the TDP-43-injected monkey brain. Further, an in vitro cleavage assay showed the increased generation of cleaved TDP-43 products by TDP-43-injected monkey brain lysates compared with GFP-injected monkey brain, and this increase could be completely inhibited by the caspase-4 specific inhibitor LEVD-fmk (Fig. 4d).

**Fig. 5:**
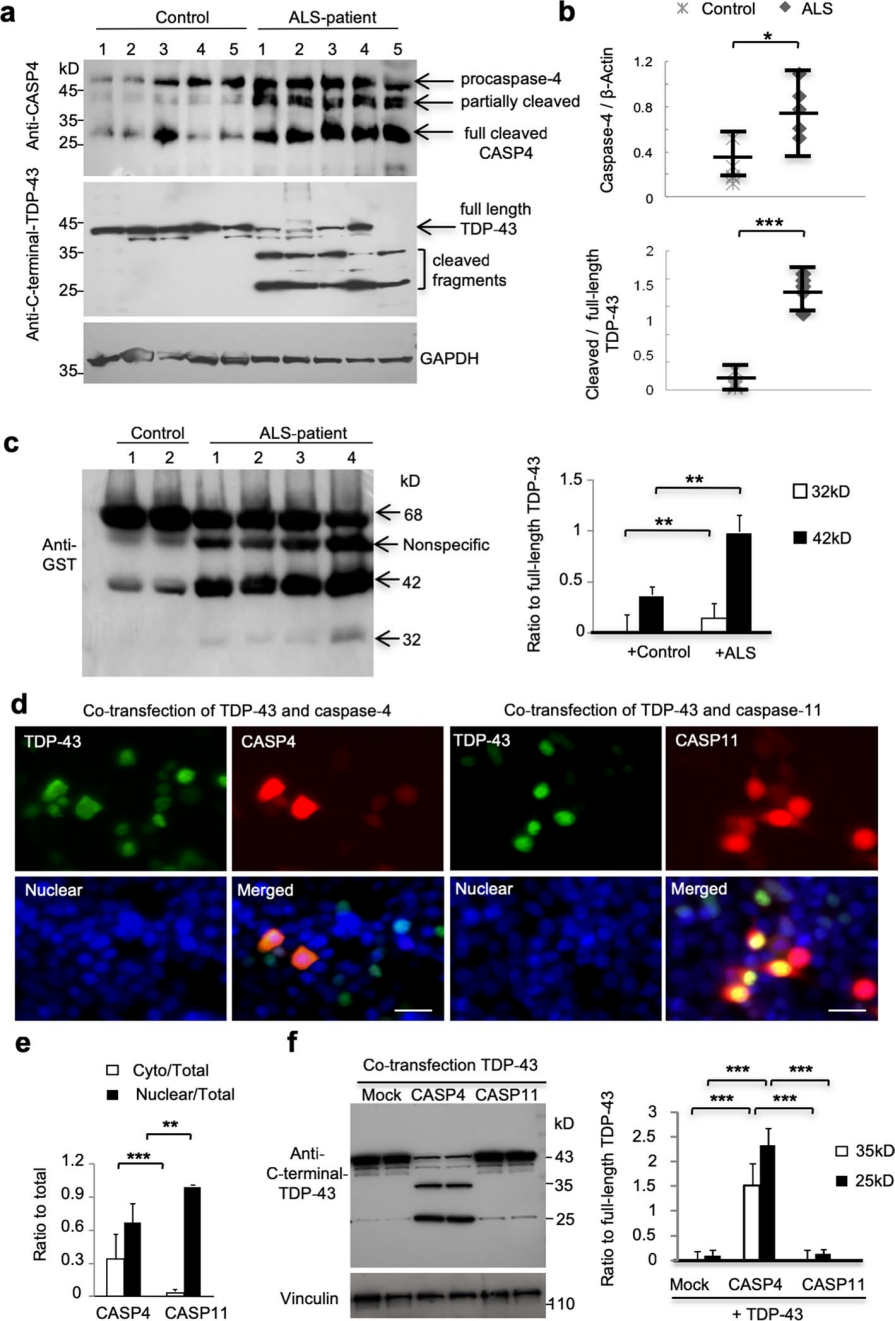
Caspase-4 is increased in ALS patient brains and mediates cytoplasmic localization of TDP-43. **a, b** Western blotting analysis of caspase-4 (CASP4) and C-terminal-TDP-43 expression in five ALS patients and five non-ALS control individuals (**a**). Ratios of activated caspase-4 bands (arrow) to GAPDH, or cleaved TDP-43 to full-length TDP-43 are presented right of the blots (**b**). *P < 0.05, ***P < 0.001. **c** Western blotting analysis of GST-TDP-43 (M337V) cleavage after incubation with the brain cortex lysates from ALS patients or non-ALS control individuals. Note that ALS patient brain lysates had much more abundant TDP-43 fragments (32- and 42-kD) than the control brain lysates. **P < 0.01. **d, e** Mouse N2A cells were transfected with pEGFP-TDP-43 (M337V) and DesRed-human caspase-4 (CASP4) or DesRed-mouse-caspase-11 (CASP11). The green fluorescent TDP-43 shows both cytoplasmic and nuclear localization in the cells co-transfected with DesRed-human-caspase-4, but not DesRed-mouse-caspase-11. The nuclei are stained by DAPI (**d**). Scale bars: 20 μm. Quantification of transfected N2A cells showing cytoplasmic (Cyto) or nuclear TDP43 over the total number of transfected cells when caspase-4 (CASP4) or caspase-11 (CASP11) was co-transfected. Counting the number of 120 positive cells under 40× lens in random six fields (**e**) (*n* = 3 independent experiments for each group, ** *P *< 0.01, *** *P *< 0.001). **f** Western blotting analysis of transfected N2A cells also showed that co-expression of caspase-4, but not caspase-11, markedly increased the generation of truncated TDP-43 (35- and 25-kD) fragments, which was also confirmed by the quantification of the ratios of truncated TDP-43 to full-length TDP-43 on the blots (right panel). *n* = 3 experiments, ***P < 0.001

A previous study reported the increased amounts of caspase-4 in the spinal cords of ALS patients [3]. Western blotting analysis of the postmortem brain cortex tissues from 5 ALS patients and 5 non-ALS individuals revealed that the activated form (between 25- and 45-kD) of caspase-4 was indeed increased in the ALS samples (Fig. 5a, Suppl. Table 1). Quantitative analysis of the ratios of the activated caspase-4 to the full-length caspase-4 or the loading control GAPDH on the same blots also confirmed the selective increase of activated caspase-4 in the ALS patient brains (Fig. 5b). The increased caspase-4 activity in postmortem ALS patient brain tissues allowed us to explore whether this increased activity is associated with the generation of TDP-43 fragments. Thus, we incubated GST-TDP-43 with postmortem brain tissue lysates. Consistently, lysates from ALS patient brains also produced more TDP-43 fragments than the brain lysates from control individuals (Fig. 5c). To verify that TDP-43 is selectively cleaved by caspase-4 but not mouse homologous caspase-11, we co-transfected pEGFP-TDP-43 (M377V) with DesRed–human caspase-4 or mouse caspase-11 in mouse N2A cell line. The green fluorescent labeling shows the cytoplasmic localization of transfected TDP-43 in N2A cells when co-transfected with human caspase-4 (red). However, mouse caspase-11 transfection was unable to cause the cytoplasmic distribution of TDP-43 (Fig. 5d, e) and to generate of the truncated TDP-43 (Fig. 5f), supporting the idea that only caspase-4 cleavage generates TDP-43 fragments that are able to accumulate in the cytoplasm.

### Caspase-4 generates cytoplasmic TDP-43 fragments

**Fig. 6:**
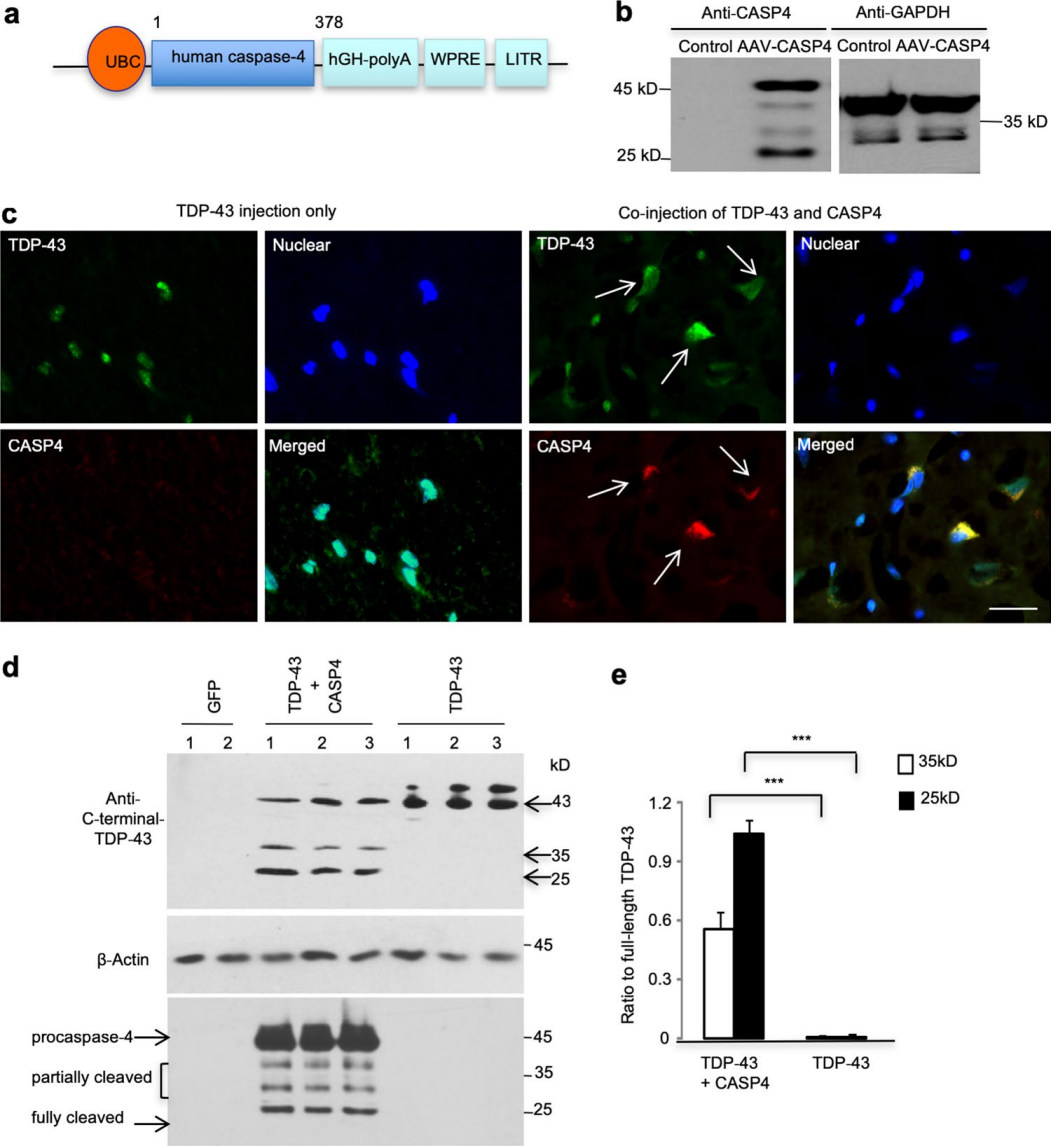
Caspase-4 cleavage of mutant TDP-43 leads to the cytoplasmic localization of TDP-43 fragments in the mouse brains. **a** Schematic diagrams of AAV9 viral vector that expresses human caspase-4 under the control of the human ubiquitin promoter. **b** Expression of caspase-4 (CASP4) in mouse N2A cells infected by AAV–human-caspase-4. Caspase-4 was detected via Western blotting. **c** The mouse brain substantia nigra was injected with AAV-TDP-43 and AAV-caspase-4. Double immunofluorescent staining with antibodies to caspase-4 and C-terminal-TDP-43 showed the cytoplasmic localization of TDP-43 (green) in cells (arrows) that also express human caspase-4 (red). In cells expressing AAV-TDP-43 only, TDP-43 is localized in the DAPI stained nuclei. Scale bar: 40 μm. **d, e** Western blotting analysis of the mouse substantia nigra tissues co-injected with AAV-caspase-4 and AAV-TDP-43(M337V) using the antibody to C-terminal-TDP-43 (upper panel). The samples were also probed with anti-caspase-4 (lower panel). AAV-GFP or AAV-TDP-43(M337V) injection alone served as controls (**d**). Note that co-expression of human caspase-4 leads to the generation of C-terminal TDP-43 fragments of 35 and 25 kD, which is supported by the quantitative data of the ratio of cleaved TDP-43 fragments to the intact form (43 kD) (**e**) (*n* = 6, ***P < 0.001)

If caspase-4 is absent in the mouse brain, but is responsible for the cytoplasmic accumulation of TDP-43, transgenic expression of caspase-4 in the mouse brain should also lead to the cytoplasmic accumulation of TDP-43. We generated AAV vector-expressing human caspase-4 under the control of the ubiquitin promoter (Fig. 6a) and confirmed its expression in cultured N2A cells (Fig. 6b). We then stereotaxically delivered this virus with AAV-TDP-43 (M337V) into the substantia nigra in 10–14 months old mice. As expected, immunofluorescent staining showed abundant nuclear aggregates of transgenic TDP-43 in the mouse brain in the absence of caspase-4. However, co-expression of caspase-4 caused mutant TDP-43 to distribute in the cytoplasm (Fig. 6c). Furthermore, Western blotting with the antibody to C-terminal TDP-43 confirmed that co-expression of caspase-4 could yield C-terminal 25-kD and 35-kD TDP-43 fragments (Fig. 6d, e).

**Fig. 7:**
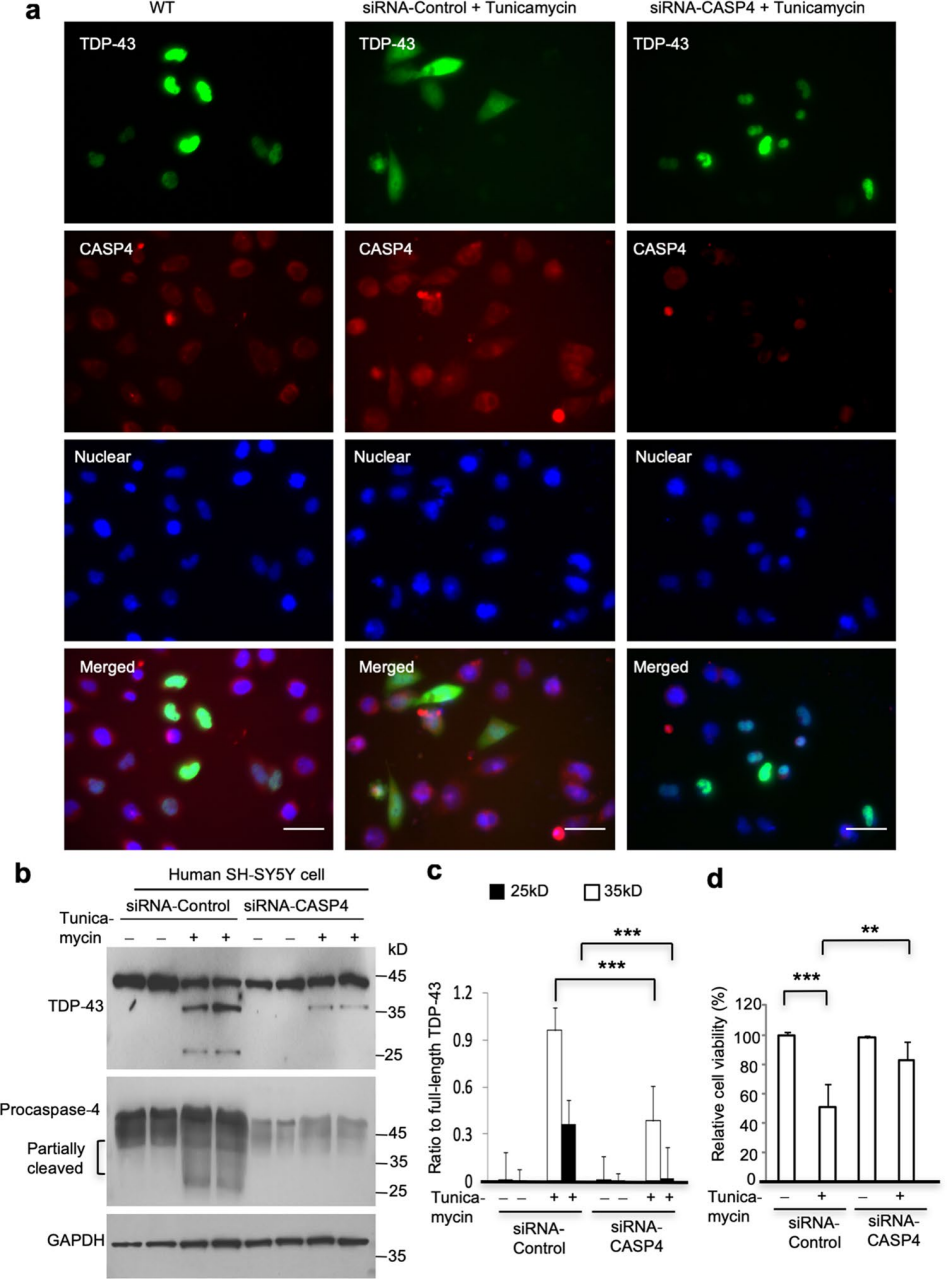
Knockdown of caspase-4 expression diminished the cytoplasmic distribution of endogenous TDP-43 in human SH-SY5Y cells. **a** Immunofluorescent staining of human SH-SY5Y cells transfected with caspase-4 (CASP4) siRNA or the control scrambled siRNA. The green fluorescent-labeled endogenous TDP-43 shows only nuclear localization when cells were not treated with tunicamycin. Tunicamycin treatment increased the cytoplasmic distribution of endogenous TDP-43, and this increase could be attenuated by siRNA-caspase-4 but not siRNA–control. The nuclei were stained by DAPI. Scale bars: 40 μm. **b** Western blotting of human SH-SY5Y cells that were transfected with caspase-4 siRNA or the scrambled control siRNA for 48 h and then treated with tunicamycin (1 μg/ml) for 12 h. The blots were probed with anti-C-terminal TDP-43 antibody. Note that tunicamycin treatment increased the level of activated caspase-4 produced from pro-caspase-4 and that siRNA-caspase-4 could inhibit this increase. **c** Quantitative analysis of the relative level (ratio to full-length TDP-43) of cleaved endogenous TDP-43 on Western blotting (**b**). The data were obtained from three independent experiments. (***P < 0.001). **d** Viability assay of SH-SY5Y cells transfected with siRNA-caspase-4 or its scrambled siRNA control and then treated with 1 μg/ml tunicamycin. The values (mean ± SEM) of cell viability are presented as  % of that of the control cells without tunicamycin/siRNA treatment and were obtained from three independent experiments. (**P < 0.01; ***P < 0.001)

To provide direct evidence for the causative role of casapse-4 in the cleavage of TDP-43 in cells, we used siRNAs to knockdown the expression of endogenous caspase-4 in cultured human neural SH-SY5Y cells. The siRNA (siCAS4-1) had higher efficiency on silencing endogenous caspase-4 than siCAS4-2 (Suppl. Figure 12). The siCAS4-1 was then transfected into SH-SY5Y cells for 48 h, and the transfected cells were treated with tunicamycin (1 μg/ml) for 12 h, which is a known ER stress activator for caspase-4 induction [18, 58]. As expected, immunofluorescent staining showed abundant cytoplasmic distribution of endogenous TDP-43 after tunicamycin treatment. Moreover, this cytoplasmic distribution was reduced by suppressing caspase-4 expression via siRNA (siCAS4-1) (Fig. 7a). Western blotting also showed that suppressing the expression of endogenous caspase-4 could significantly reduce the generation of C-terminal 25-kD and 35-kD TDP-43 fragments (Fig. 7b, c). Tunicamycin has been reported to increase caspase-4 activity [18, 58] and reduce the viability of SH-SY5Y cells [16, 61]. We therefore measured cell viability of the cultured SH-SY5Y cells using CCK-8, a widely used assay that can sensitively measure the viability of a variety of cultured cells including SH-SY5Y cells [10, 56]. We found that inhibition of caspase-4 via siRNA-caspase-4 also significantly increased cell viability after tunicamycin treatment in parallel with reduction of cytoplasmic accumulation of endogenous TDP-43 (Fig. 7d).

**Fig. 8:**
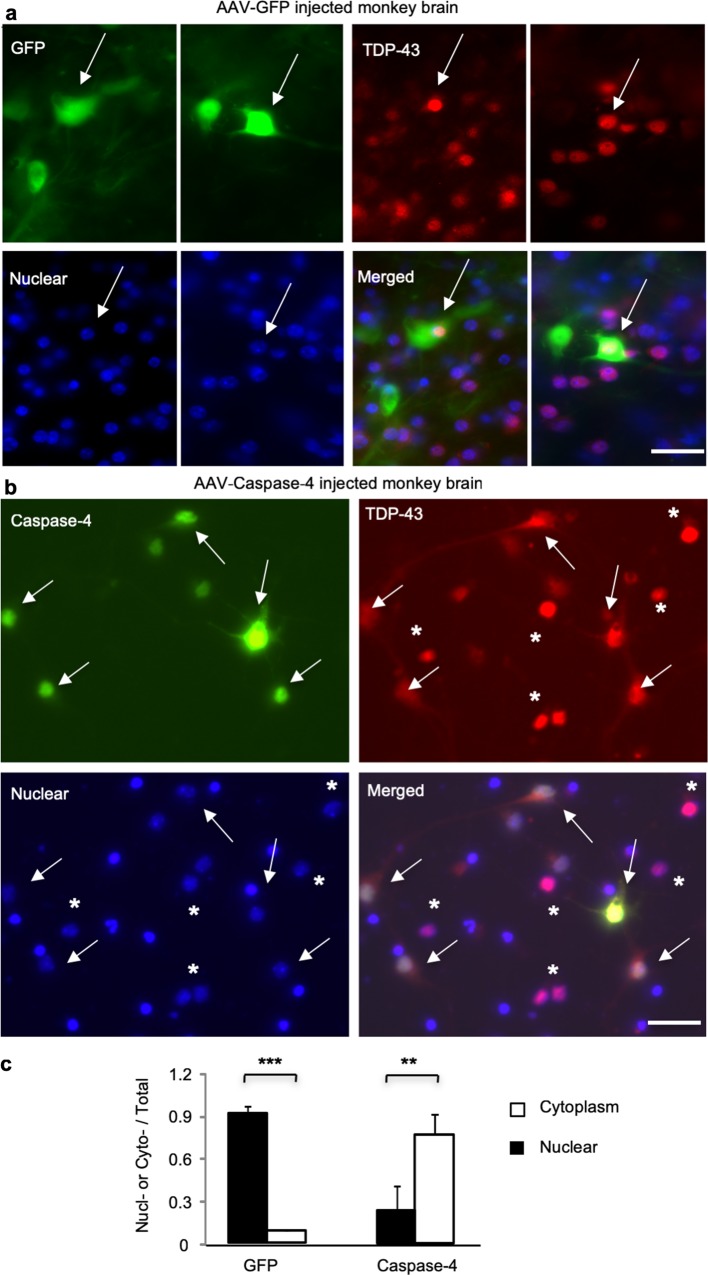
Caspase-4 cleavage leads to the cytoplasmic distribution of monkey endogenous TDP-43 in vivo. The monkey brain prefrontal cortex was injected with AAV-GFP (control) (**a**) or AAV-caspase-4 (**b**). Double immunofluorescent staining with antibodies to C-terminal TDP-43 and GFP or caspase-4 showed the nuclear localization of endogenous TDP-43 (red) in cells (arrows) that express GFP (green). However, in cells expressing AAV-caspase-4, endogenous TDP-43 is localized in the cytoplasm as compared to those that do not express caspase-4. DAPI stained nuclei (star). Scale bars: 40 μm. **c** Quantitative analysis of the relative numbers of cells expressing nuclear (Nucl-) or cytoplasmic (Cyto-) TDP-43 over the total number of TDP-43-containing cells. The data (% of cells with nuclear or cytoplasmic TDP-43) are mean ± SEM and obtained by counting 80 cells in 6 random images (40× lens) in the injected area per sample from three monkeys (**P < 0.01, ***P < 0.001)

**Fig. 9:**
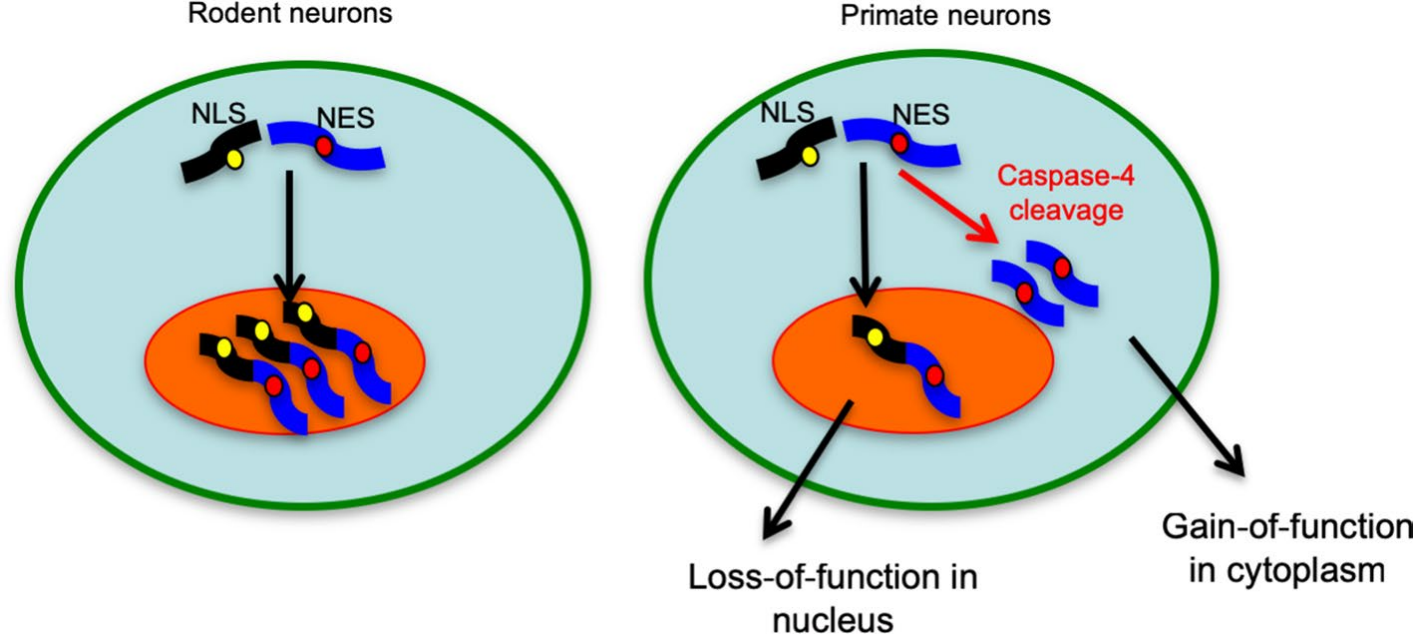
A proposed model for differential subcellular accumulation of TDP-43 in rodent and primate brains. The unique expression of caspase-4 in the primate brains causes the cleavage of full-length TDP-43 and generation of its C-terminal fragments that that can accumulate in the cytoplasm. While this cytoplasmic redistribution can reduce the level of nuclear TDP-43 to cause loss-of-function, mutations in the C-terminal region of TDP-43 could promote the misfolding and accumulation of C-terminal TDP-43 in the cytoplasm to elicit a toxic gain-of-function

If TDP-43 is cleaved by activated caspase-4, increased activity or expression of caspase-4 should promote the cytoplasmic distribution of endogenous TDP-43 in the monkey brain. To test this idea, we injected the same titer of AAV-caspase-4 or control AAV-GFP into the prefrontal cortex in 11-year-old wildtype monkeys. After 3 weeks, the injected brain prefrontal cortex was isolated for immunofluorescent staining with antibodies to caspase-4 and C-terminal TDP-43. Double immunofluorescent staining showed that endogenous TDP-43 in the normal monkey brain is predominantly distributed in the nucleus (Fig. 8a). However, in the AAV-caspase-4 injected monkey brain region, endogenous TDP-43 shows the cytoplasmic and diffuse distribution neuronal cells (Fig. 8b, c). This cytoplasmic distribution is unlikely due to stereotaxic injection and overexpression of AAV vector, as quantitative data show that injection of AAV-GFP was unable to alter the distribution of endogenous TDP-43 in the nucleus (Fig. 8a, c). Because caspase-4 is expressed in the primates but not rodents, we propose that caspase-4 mediates the unique cytoplasmic accumulation of mutant TDP-43 in primates including humans (Fig. 9).

## Discussion

Mislocalization of TDP-43 in the cytoplasm and loss of its nuclear distribution are the major pathological hallmarks in ALS and FTLD [2, 15, 35] and other neurological disorders [8, 9, 17, 23, 33, 48]. Thus, the classification of TDP-43 proteinopathy is a combination of cytoplasmic inclusions and nuclear depletion of TDP-43 [30], which lead to gain-of-function and loss-of-function, respectively [28, 47, 57]. Although it is known that the abnormal level of TDP-43 is critical for developing neuropathology, the subcellular distribution of mutant TDP-43 appears to be independent of the levels of TDP-43 but is essentially regulated by species-related factors. This is because transgenic rodent models that overexpress either normal or mutant TDP-43 also show the predominantly nuclear accumulation of TDP-43 [14, 19, 43, 52]. This fact led us to overexpress mutant TDP-43 in the mouse and monkey brains via viral vector injection to explore the mechanism underlying the cytoplasmic accumulation of TDP-43. Using non-human primates, we found that caspase-4 cleaves TDP-43 to remove N-terminal fragments that carry the nuclear import signal, resulting in the cytoplasmic accumulation of C-terminal fragments in the primate brains.

TDP-43 is a major component of cytoplasmic aggregates in the brains and spinal cords of nearly all patients (~ 97%) with ALS and in ~ 45% of FTLD cases [2, 7, 22, 35, 40]. In addition, 57% of Alzheimer’s disease cases and some dementia patients with Lewy bodies also show TDP-43 proteinopathies in their brains [9, 17, 23, 33, 48]. However, only < 5% of ALS patients carry mutations in TDP-43 [8, 24, 27, 41]. Thus, pathological conditions other than TDP-43 mutations are the major factors responsible for the cytoplasmic accumulation of TDP-43 and that mutations in TDP-43 may exacerbate this abnormal redistribution.

Recent studies suggest that impaired nuclear–cytoplasmic transport contributes to ALS. The mutations in C9orf72 mRNA, which can also cause ALS by impairing the nuclear–cytoplasmic transport [13, 62], can affect the nuclear import of TDP-43 [25]. The idea for the impaired nuclear transport is also well supported by the abnormal cytoplasmic distribution of TDP-43 in the transgenic mice expressing a defective nuclear localization signal [20]. Although these studies clearly show that the nuclear–cytoplasmic transport plays an important role in ALS pathogenesis, it remains unclear why the predominant cytoplasmic accumulation of TDP-43 does not occur in the rodent brains. However, expression of wildtype TDP-43 in spinal cords of cynomolgus monkeys by injecting AAV vector leads to the cytoplasmic distribution of TDP-43 [49], and transgenic pig model expresses mutant TDP-43 in the cytoplasm [51]. All these differences indicate that the cytoplasmic distribution of mutant TDP-43 is species dependent.

In the current studies using non-human primates, we identified four lines of evidence supporting caspase-4 as a critical contributor to the cytoplasmic accumulation of TDP-43 in the primate brains. First, caspase-4 is found in non-human primates and humans, but not in mice. Second, caspase-4 cleaves TDP-43, but its mouse homologue caspase-11 does not. Third, co-expression of caspase-4 with mutant TDP-43 in the mouse brain leads to the cytoplasmic redistribution of TDP-43. Lastly, overexpression of caspase-4 can increase the cytoplasmic distribution of endogenous TDP-43 in the monkey brain, whereas suppressing caspase-4 expression can reduce the distribution of endogenous TDP-43 in the cytoplasm in cultured human neural cells.

In vitro studies demonstrated that TDP-43 was initially cleaved after Asp174 by caspase-4 [29], which can result in C-terminal fragments that retain NES at amino acid position (239-250) and delete NLS at amino acids (82-98). Because C-terminal TDP-43 can interact with many partners, mutations in the C-terminal TDP-43, which are frequently found in ALS patients, can facilitate the misfolding, aggregation, and abnormal interactions of truncated TDP-43 with other cytoplasmic proteins, resulting in a gain-of-toxicity function. Meanwhile, caspase-4 mediated the fragmentation of TDP-43 and their cytoplasmic redistribution can also deplete the nuclear full-length TDP-43, leading to a loss-of-function in the nucleus.

It should be pointed out that overexpression of TDP-43 can only partially mimic ALS pathology that is caused by endogenous mutant TDP-43. Similarly, neurodegeneration in TDP-43 transgenic mice may not depend on TDP-43 cleavage and is mediated by different mechanisms. The critical role of nuclear TDP-43 in gene transcription and RNA processing is well documented [12, 39, 47]. Overexpression or depletion of TDP-43 in the nucleus is known to severely affect gene expression and to cause severe phenotypes of mice [14, 38]. When considering the neuropathology and phenotypes of humans that express mutant TDP-43 at the endogenous level, the cytoplasmic accumulation of TDP-43 must be accounted for.

Our studies indicate that caspase-4 plays a critical role in the cytoplasmic accumulation of TDP-43. The relevance of this finding to other pathological conditions is also supported by the regulation of caspase-4 activity and expression. Caspase-4 is an endoplasmic reticulum (ER) membrane-bound enzyme and is activated under ER stress [18], which can be trigged by protein misfolding, aging, oxidative stress, and many environmental insults [32, 36, 37]. Our studies also show that caspase-4 is increased in ALS patient brains and the AAV-TDP-43-injected monkey brains, consistent with the early findings that caspase-4 and markers of ER stress are up-regulated in the spinal cords of patients with sporadic ALS [3, 21]. When TDP-43 is expressed at the endogenous level, caspase-4 activation and cleavage can reduce the nuclear distribution of full-length TDP-43 and increase the cytoplasmic accumulation of TDP-43, resulting in both nuclear and cytoplasmic toxicity. Thus, any pathological condition that upregulates caspase-4 activity or expression is likely to cause the cytoplasmic distribution of truncated TDP-43 and subsequent reduction in the nuclear distribution of full-length TDP-43. In support of this idea, suppressing caspase-4 expression was found to diminish the cytoplasmic distribution of TDP-43 and to improve cell viability in human neural cells after treatment with an ER stress activator. Our findings also suggest that pharmacological interventions of the abnormal cytoplasmic redistribution or accumulation of TDP-43 via altering caspase-4 activity could be a potential therapeutic strategy.

## Electronic supplementary material

Below is the link to the electronic supplementary material.

Supplementary material 1 (PDF 1272 kb)

Supplementary material 2 (MP4 489 kb)

## Abbreviations

AAV: Adeno-associated virus
ALS: Amyotrophic lateral sclerosis
FTLD: Frontotemporal lobar degeneration
TDP-43: Transactive response DNA-binding protein 43
