# Supplementary material for: Caspase-4 mediates cytoplasmic accumulation of TDP-43 in the primate brains
Source: Acta Neuropathol. 2019 Feb 27;137(6):919–37. doi: 10.1007/s00401-019-01979-0 (PMC6531422; doi:10.1007/s00401-019-01979-0)
Supplement: Supplementary file 1 — Supplementary material 1 (PDF 1272 kb) [file 401_2019_1979_MOESM1_ESM.pdf]

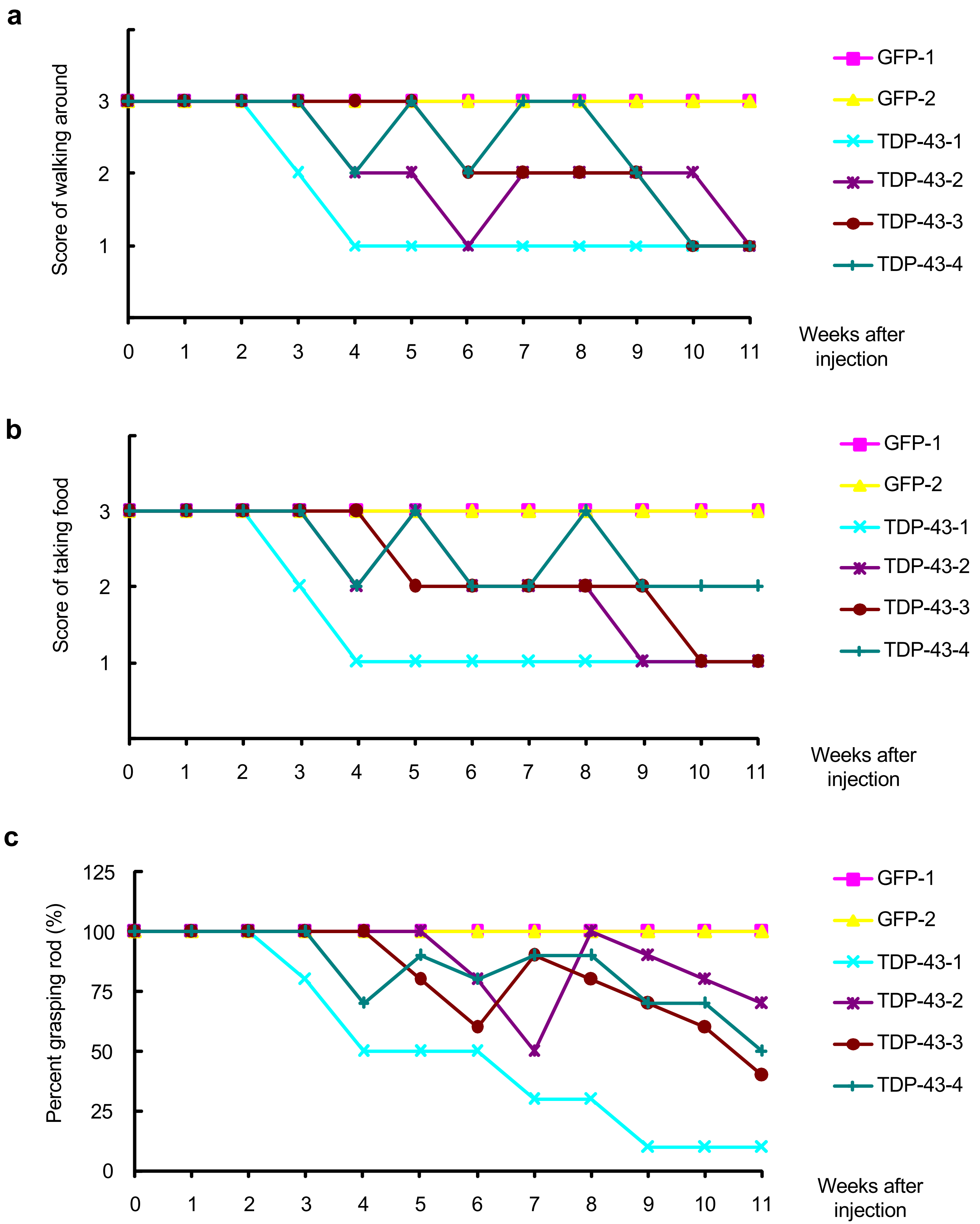
a
Score of walking around
Weeks after injection
b
Score of taking food
Weeks after injection
c
Percent grasping rod (%)
Weeks after injection
GFP-1
GFP-2
TDP-43-1
TDP-43-2
TDP-43-3
TDP-43-4

Supplemental Fig. 1 Reduced movements and upper limb grasping in monkeys injected with AAV-TDP-43 in the right side of the substantia nigra. The scores of walking around (**a**) and using the left upper hand for taking food (**b**), and the frequency of grasping a rod (**c**). The animals were examined six times on the same day in different weeks after injection of AAV-TDP-43. AAV-GFP injection served as a control. A score of 3 indicates that the monkeys can freely walk around or take food using either the left or right hand; the score 2 indicates that monkeys had difficulty walking or grasping when the left hand was used, and frequently used the right hand to take food; The score 1 indicates that monkeys lost the support from the left hand when walking, and could not raise the left hand to take food or grasp the ceiling fence or a test rod.

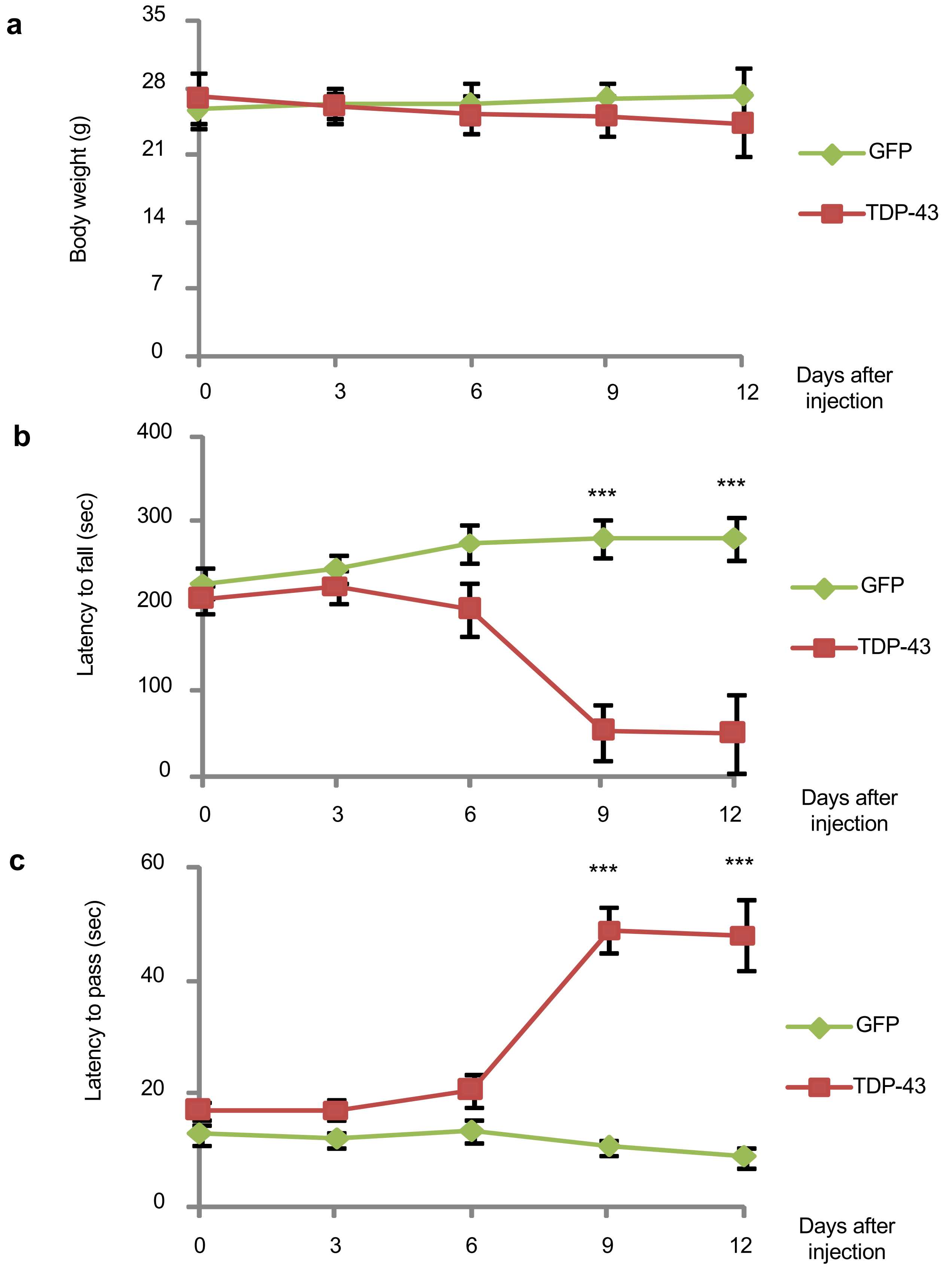

Supplemental Fig. 2 Reduced motor function in mice injected with AAV-TDP-43 in the right side of the substantia nigra. The body weight (**a**), rotarod performance (**b**), and balance beam test (**c**) were recorded at different days after injection. AAV-GFP injection served as a control (n=12 mice per group for AAV-GFP and TDP-43 injection, *** *P*<0.001).

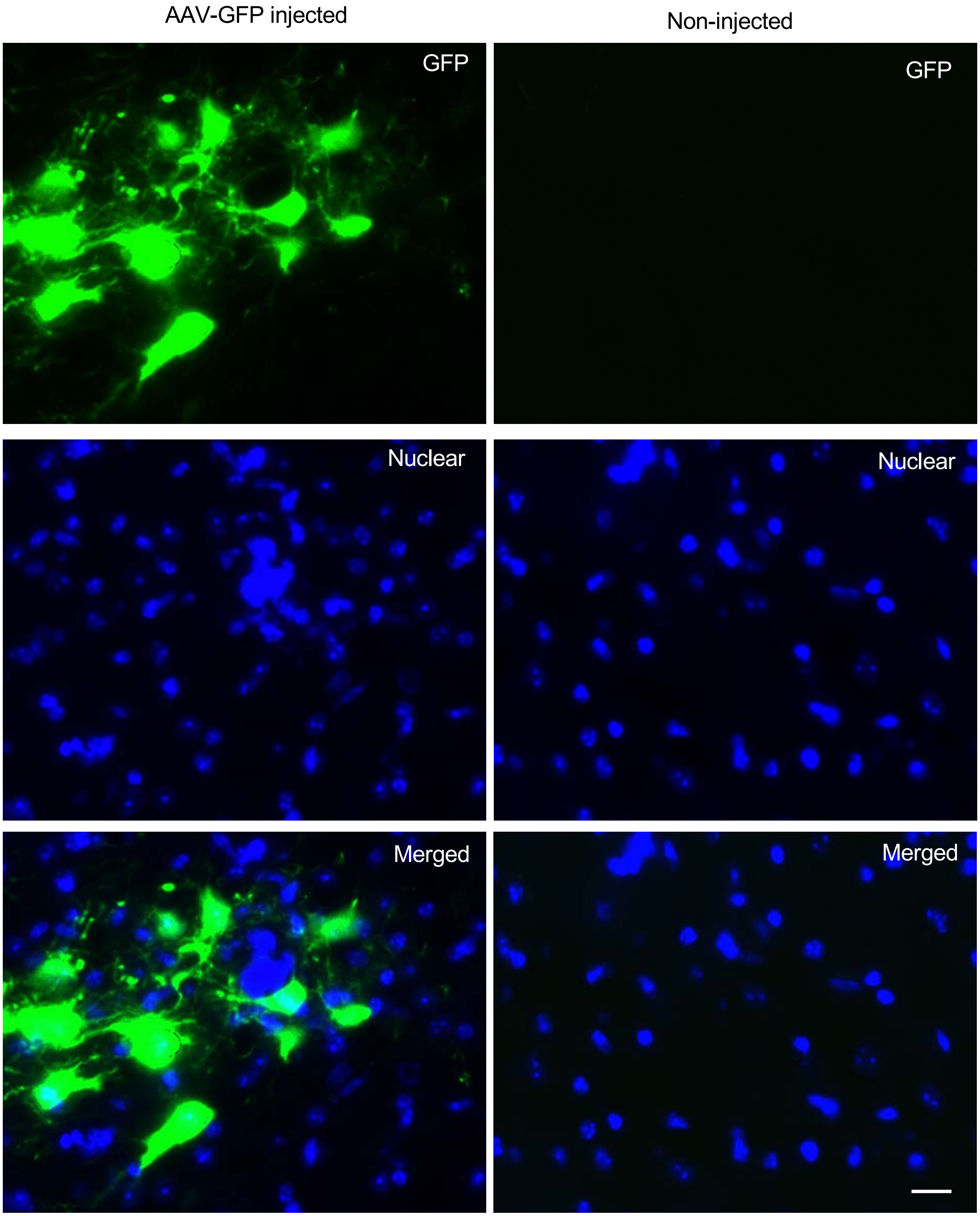

Supplemental Fig. 3 Immunostaining of the monkey substantial nigra injected with AAV-GFP. Control: non-injection side. The nuclei were stained with DAPI. Scale bar: 10 μm.

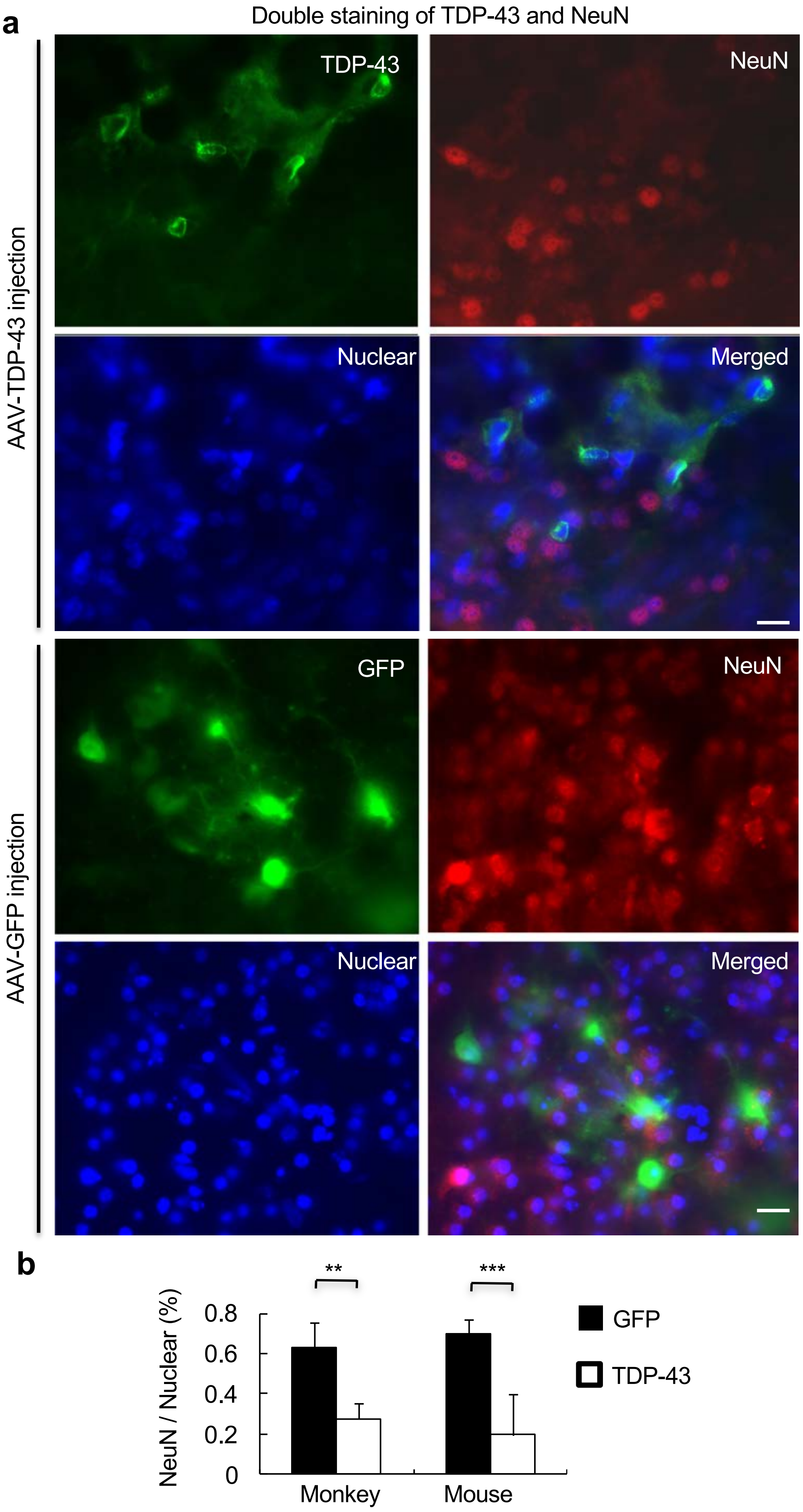
a
Double staining of TDP-43 and NeuN
AAV-TDP-43 injection
TDP-43
NeuN
Nuclear
Merged
AAV-GFP injection
GFP
NeuN
Nuclear
Merged
b
NeuN / Nuclear (%)
0.8
0.6
0.4
0.2
0
**
***
Monkey
Mouse
GFP
TDP-43

Supplemental Fig. 4 Double-immunostaining of monkey substantial nigra injected with AAV-TDP-43. **a** the injected monkey brain was injected using antibodies to TDP-43 and NeuN. The nuclei were stained with DAPI. Scale bars: 10 μm. **b** Counting the relative number of NeuN-positive cells per image (40X lens) in the injected monkey or mouse striatum. The results were obtained by counting a total of 120 cells in random six images for each group.

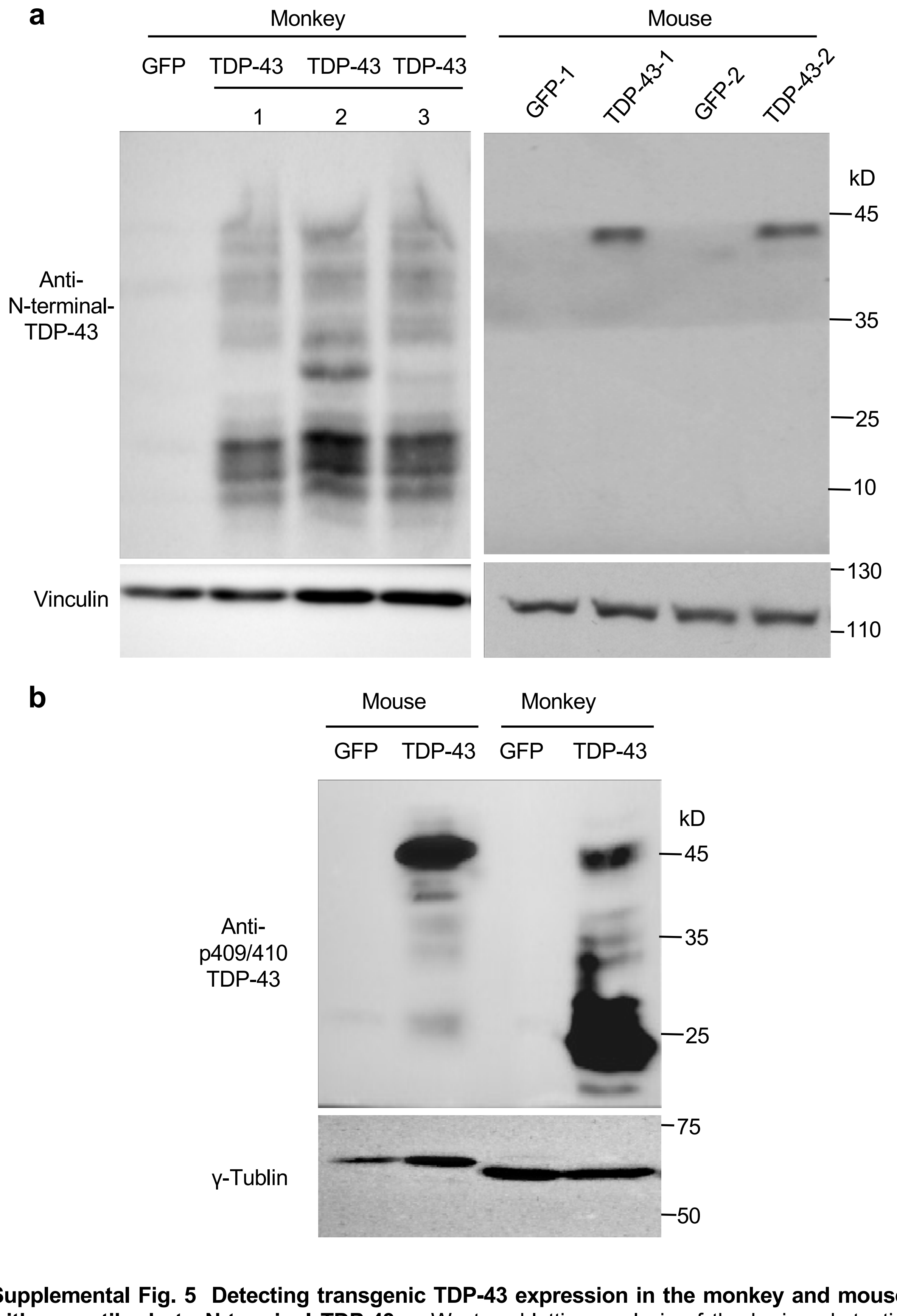

Supplemental Fig. 5 Detecting transgenic TDP-43 expression in the monkey and mouse brain with an antibody to N-terminal TDP-43. a Western blotting analysis of the brain substantial nigra tissues of several TDP-43 monkeys and mice that were injected with AAV-GFP or AAV-TDP-43. The blots were probed with the antibody to N-terminal-TDP-43 (1-261 amino acids). **b** Detection of phosphorylated TDP-43 using anti-p409/410 TDP-43 via western blotting. Note that the injected monkey substantial nigra tissues generated much more truncated TDP-43 than the injected mouse tissues.

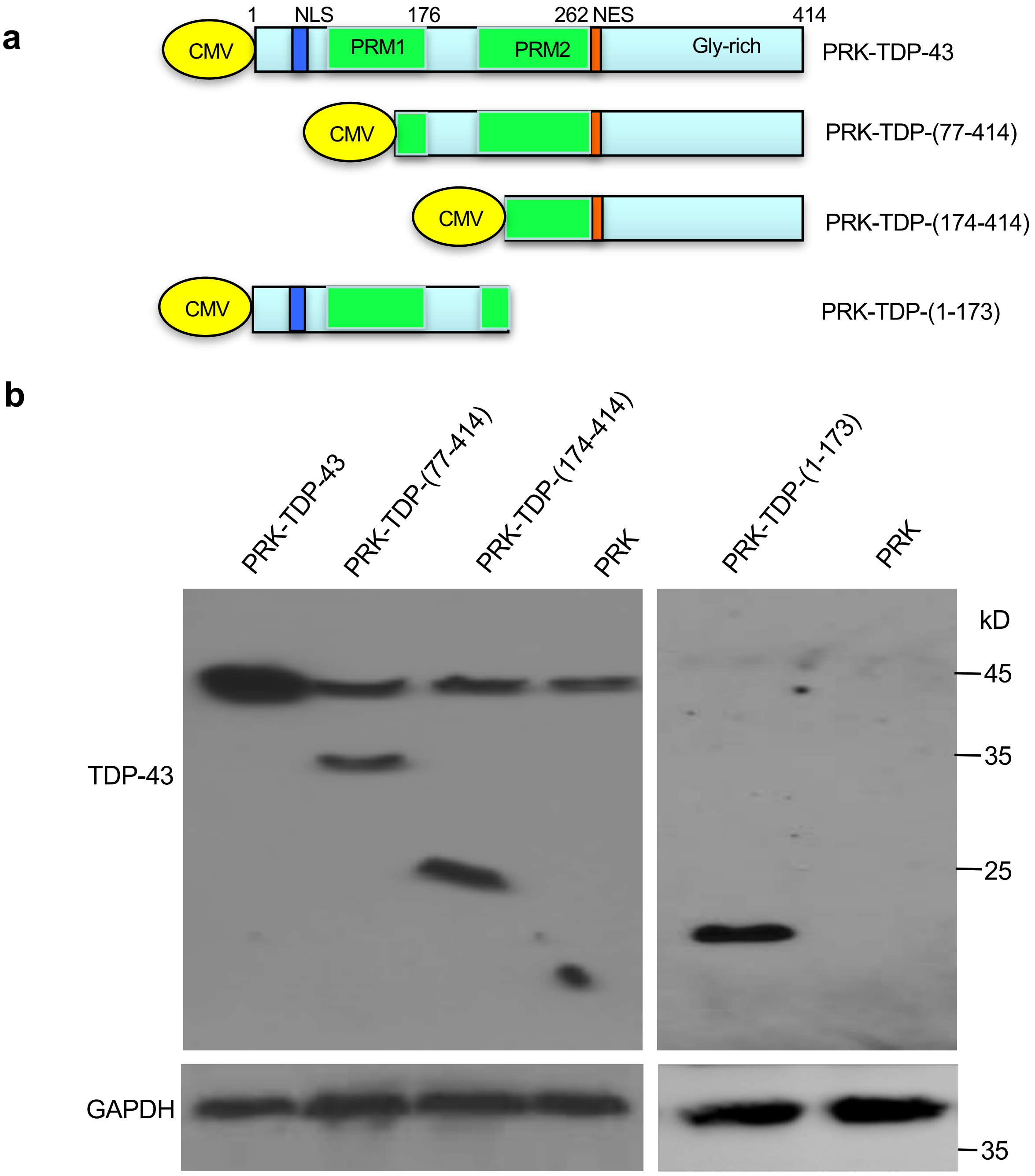

Supplemental Fig. 6 Generation of vectors expressing TDP-43 C-terminal or N-terminal fragments. a The plasmid DNA structure for expressing full length TDP-43, truncated TDP-(77-414), C-terminal TDP-(174-414), or N-terminal TDP-(1-173) under the CMV promoter in PRK vector. **b** The expression of PRK-TDP-43, PRK-TDP-(77-414), PRK-TDP-(174-414) and PRK-TDP-(1-173) in transfected N2A cells was detected via western blotting.

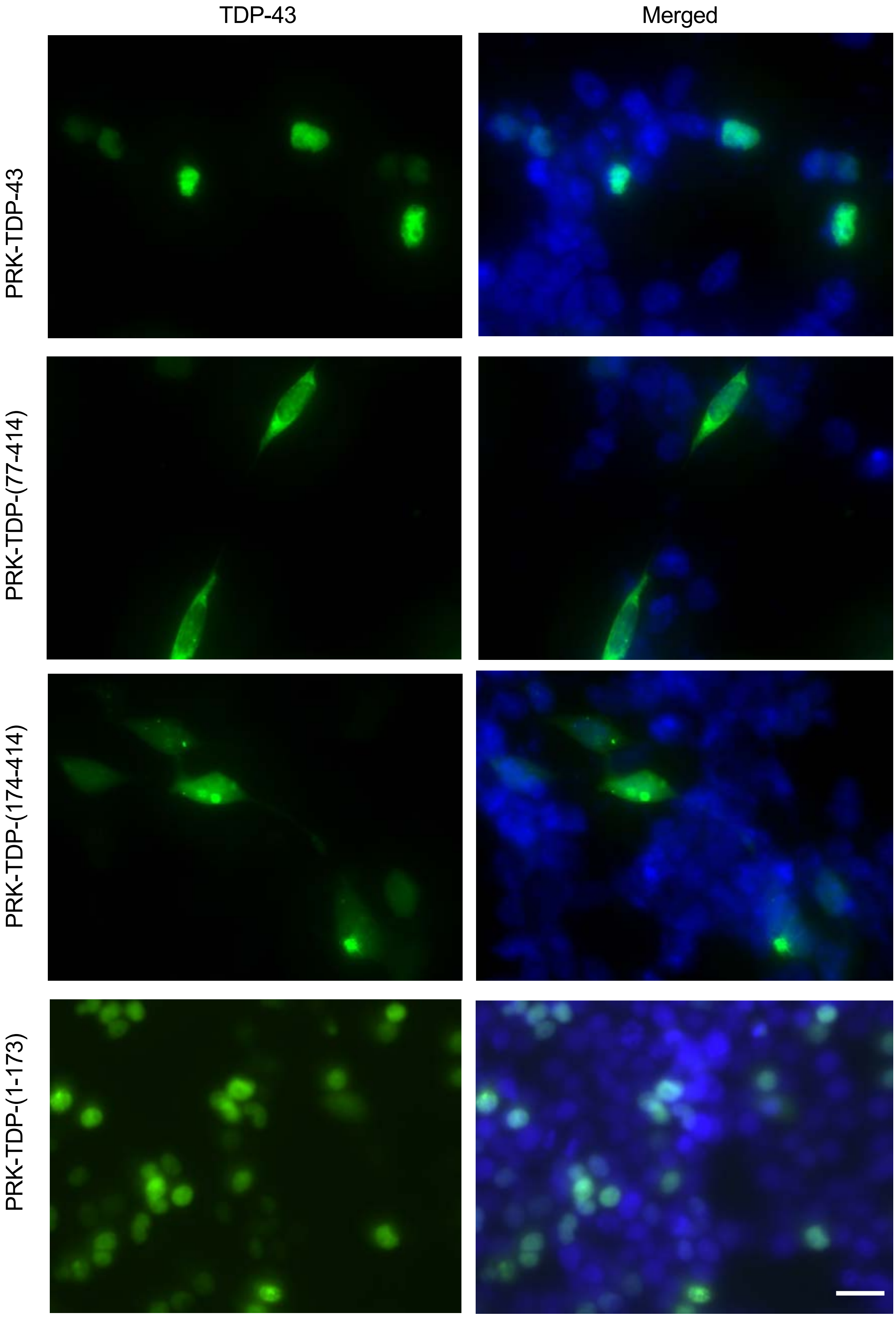

Supplemental Fig. 7 Cellular localization of the different TDP-43 fragments. Immunostaining of mouse N2A cells transfected with PRK-TDP-43, PRK-TDP-(77-414), PRK-TDP-(174-414) or PRK-TDP-(1-173) using antibodies that recognized N-terminal (1-261 amino acids) or C-terminal peptide corresponding to residues surrounding Gly400 of TDP-43. The nuclei were stained with DAPI. Scale bar: 10 μm.

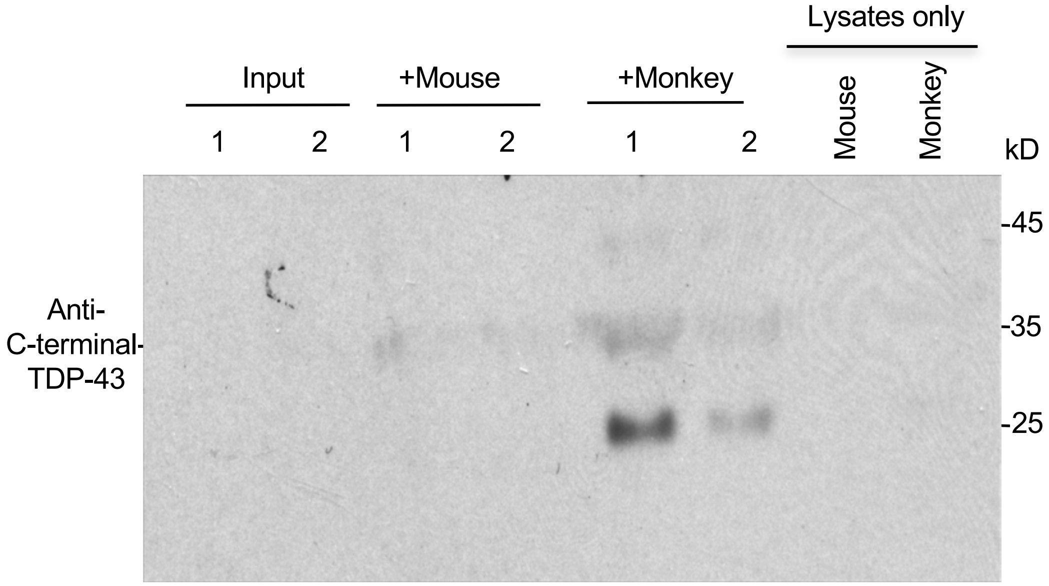

Supplemental Fig. 8 Detecting C-terminal TDP-43 fragments generated by GST-TDP-43 in the *in vitro* caspase-4 cleavage assay. Western blotting analysis of GST-TDP-43(M337V) that was incubated with brain homogenate lysate from mouse or monkey brain striatum. The supernatant after precipitating GST-TDP-43 beads was detected by anti-C-terminal-TDP-43 via western blotting.

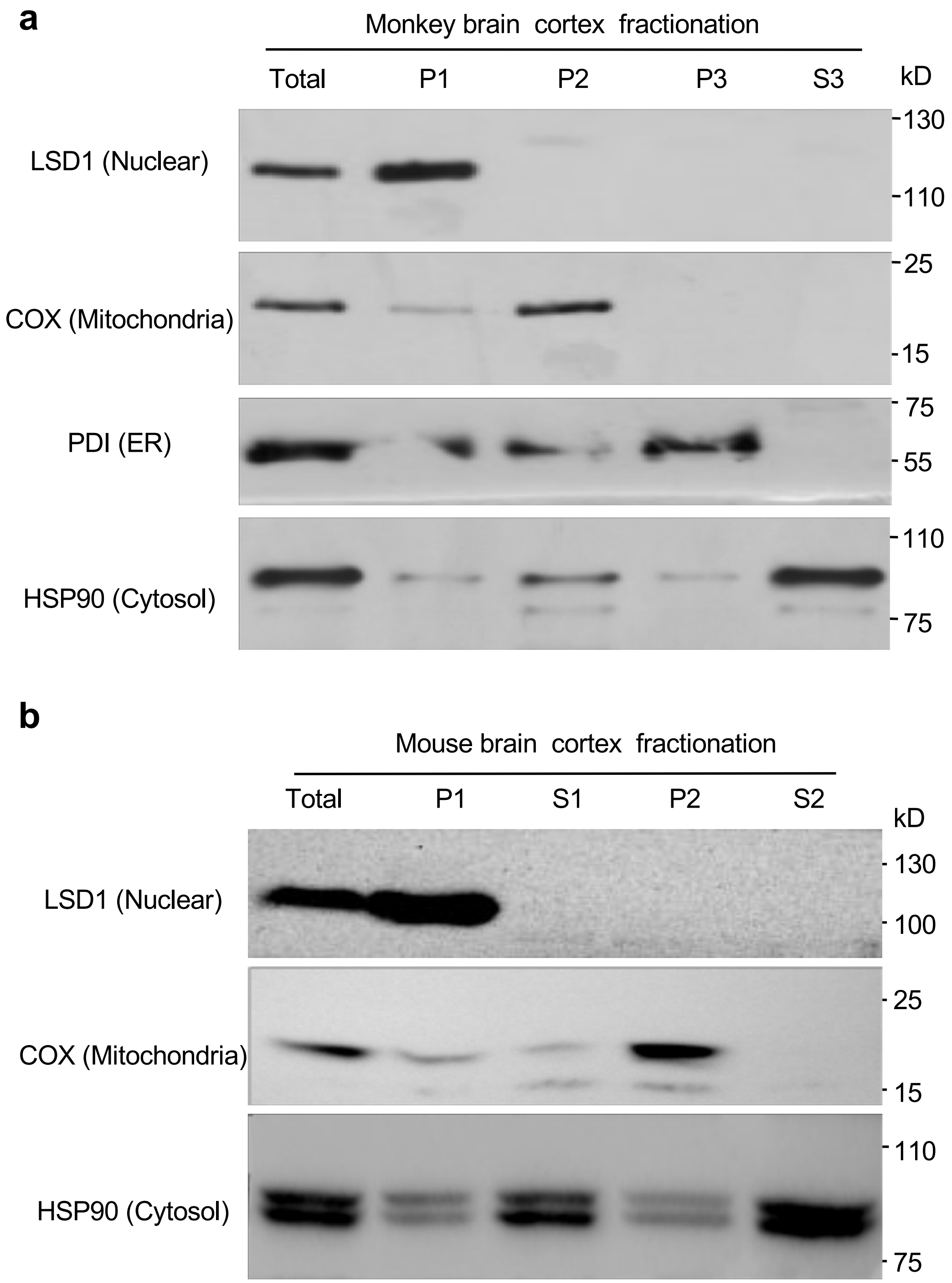

Supplemental Fig. 9 Subcellular fractionation of the cortex of rhesus monkeys (a) and mice (b). Western blotting with antibodies to different organelle marker proteins revealing P1, P2, P3 and S3 fractions, which are enriched in different subcellular marker proteins. LSD1: nuclear lysine-specific demethylase 1; COX: mitochondrial complex IV of cytochrome c oxidase subunits; PDI: protein disulfide isomerase in endoplasmic reticulum; HSP90: heat shock protein 90 in cytoplasm.

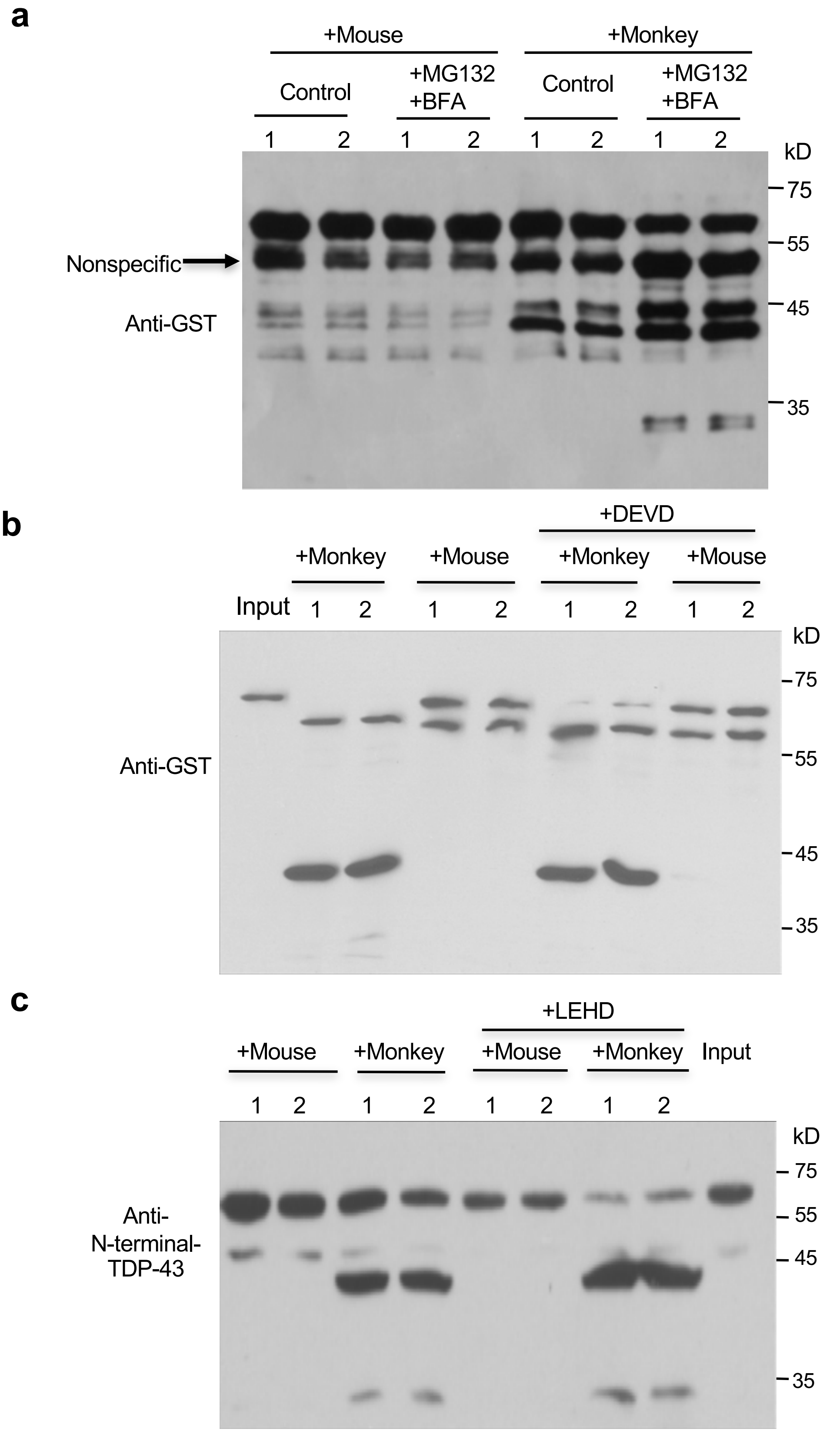

Supplemental Fig. 10 Inhibition of the UPS and autophagy did not prevent but increased the generation of fragmented TDP-43. a Western blotting analysis of cleaved TDP-43 fragments generated by the monkey brain lysates, MG132 (10 μM) and BFA (5 μg/ml) were added to the lysates for 16 h or during incubation, and this treatment increased the levels of cleaved TDP-43 by the monkey brain lysates. The blot was probed with antibody to N-terminal TDP-43. **b, c** *In vitro* cleavage assays of TDP-43 with caspase inhibitors. Western blotting analysis of GST-TDP-43(M337V) that was incubated with mouse or monkey brain striatal lysates. The caspase-3 and -7 inhibitor DEVD-fmk (**b**) or caspase-9 inhibitor LEHD-fmk (**c**) was added in the lysates but was unable to block the cleavage of TDP-43. Protein expression was detected by anti-GST or anti-N-terminal-TDP-43.

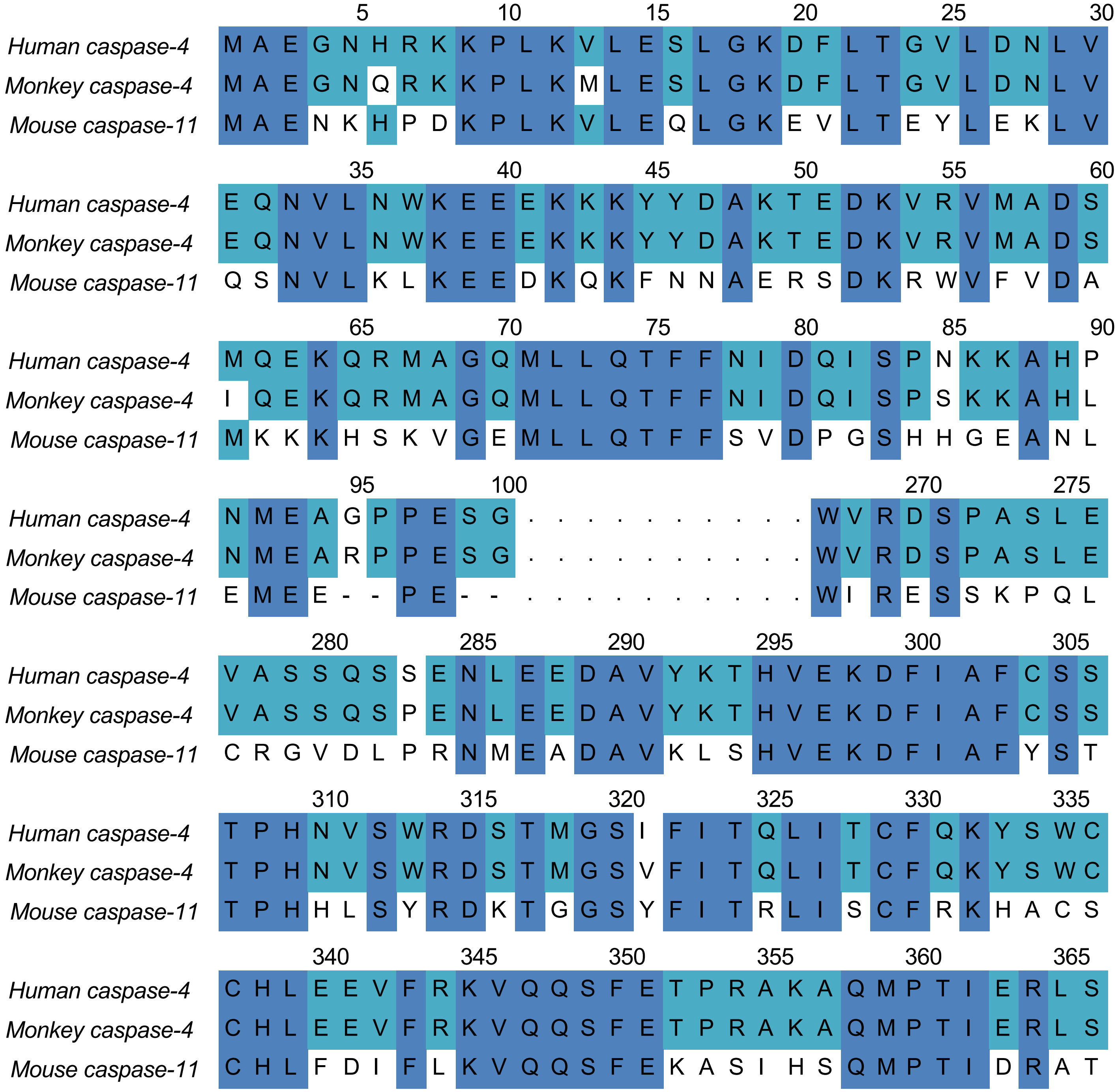

Supplemental Fig. 11 Comparison of amino acid sequences of human caspase-4, monkey caspase-4, and mouse caspase-11.

a

siRNA for caspase-4	Target sequence
siCAS4-1	GUGUAGAUGUAGAAGAGAAtt
siCAS4-2	AAGUGGCCUCUUCACAGUCAUtt

b

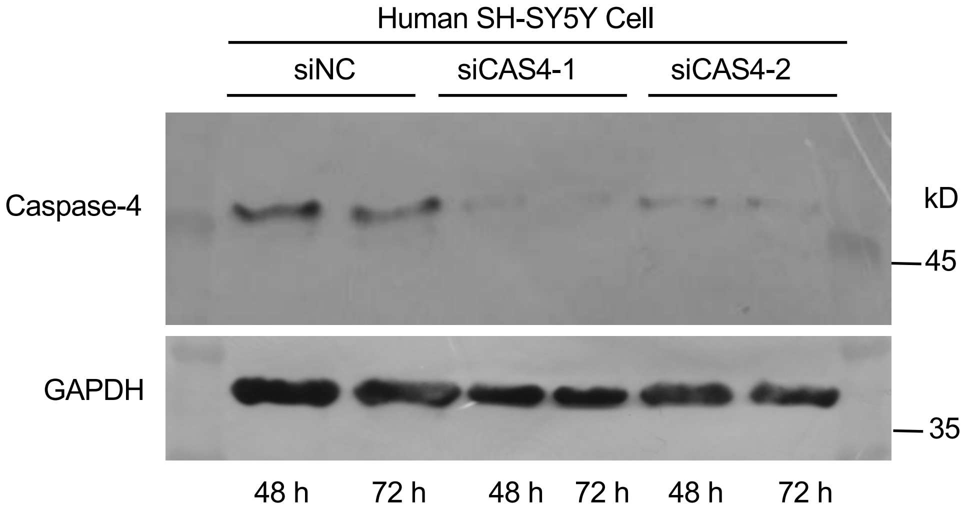

Supplemental Fig. 12 Suppressing endogenous caspase-4 via siRNA in human SH-SY5Y cells. **a** The target sequences of siRNA to caspase-4. **b** Western blotting analysis of human SH-SY5Y cells transfected after 48 h or 72 h showing that knockdown of caspase-4 via siRNA (siCAS4-1 or siCAS4-2) decreased the expression of endogenous caspase-4. Control is a scramble siRNA (siNC).

Supplemental Table S1 Postmortem brain tissues used for investigation

CASE	Case Number	Frozen Tissue Provided	Paraffin Sections Provided	PMI (hr)	Age at Onset	Age at Death	Duration	ApoE	Race /Sex
Control	E06-45	F	F	6.5		46		E3/3	wf
Control	E06-114	F	F	6.5		53		E4/4	bm
Control	E08-101	F,sp cd	F,sp cd	11.5		78		E3/3	wf
Control	E08-137	F,sp cd	F,sp cd	15.5		92		E3/3	wf
Control	E10-142	F,sp cd	F,sp cd	5.5		94		E3/3	wm
ALS	E04-56	F	F,sp cd	9.5	70	71	~1	E2/3	wm
ALS	E08-86	F	F,sp cd	13	71	71	6 mos	E3/3	wm
ALS	E09-35	F,sp cd	F,sp cd	6	63	67	3.5	E3/3	wf
ALS	E11-75	F,sp cd	F,sp cd	11	66	68	2	E3/3	wm
ALS	E11-81	F,sp cd	F,sp cd	12	69	74	5	E3/3	wf
